# G2DR: a genotype-first framework for genetics-informed target prioritization and drug repurposing

**DOI:** 10.1093/bib/bbag427

**Published:** 2026-08-10

**Authors:** Muhammad Muneeb, David B Ascher

**Affiliations:** School of Chemistry and Molecular Biology, The University of Queensland, Queen Street, 4067 Queensland, Australia; Computational Biology and Clinical Informatics, Baker Heart and Diabetes Institute, Commercial Road, 3004 Victoria, Australia; School of Chemistry and Molecular Biology, The University of Queensland, Queen Street, 4067 Queensland, Australia; Computational Biology and Clinical Informatics, Baker Heart and Diabetes Institute, Commercial Road, 3004 Victoria, Australia

**Keywords:** drug repurposing, human genetics, TWAS, genetically predicted expression, target prioritization, translational bioinformatics

## Abstract

Human genetics offers a scalable route to therapeutic discovery, but practical frameworks that convert genotype-derived signal into ranked target and drug hypotheses remain limited, particularly when matched disease transcriptomics are unavailable. We present G2DR, a genotype-first computational prioritization framework that integrates genetically predicted gene expression, multi-method gene-level testing, pathway enrichment, network context, druggability, and multi-source drug–target evidence to generate hypotheses for downstream follow-up. In a migraine case study of 733 UK Biobank participants (53 cases, 680 controls) using stratified five-fold cross-validation, G2DR imputed genetically regulated expression across seven transcriptome-weight resources and ranked genes using a reproducibility-aware discovery score derived only from training and validation data, followed by a balanced integrated score for target selection. Internal held-out evaluation within the same UK Biobank-derived analytical framework achieved gene-level ROC–AUC of 0.775 and PR-AUC of 0.475 for recovery of test-significant genes, while retaining enrichment for curated migraine-associated biology. Mapping prioritized genes to compounds through Open Targets, DGIdb, and ChEMBL produced drug sets enriched for migraine-linked and literature-associated compounds relative to a global drug background. However, tiered benchmarking showed limited recovery of migraine-specific approved therapies, with stronger signal from mechanism-linked, off-label, and literature-associated pharmacological space. Directionality filtering further distinguished broadly recovered compounds from those with stronger mechanistic compatibility. G2DR is, therefore, best viewed as a modular framework for genetics-informed hypothesis generation in genotype-first settings, not as a clinically actionable target-identification or drug-recommendation system. Prioritized genes and compounds require independent validation.

## Introduction

Drug repurposing remains an attractive strategy for accelerating therapeutic development because existing compounds benefit from prior pharmacological, toxicological, and manufacturing knowledge [1–3]. Over the past two decades, computational repurposing has expanded from expression-signature matching [4] and network medicine [5–7] to machine-learning and knowledge-graph approaches that integrate heterogeneous biomedical evidence at scale [8, 9]. Yet many current pipelines depend on disease-specific transcriptomic profiles [10, 11], curated disease modules [6, 12], or historical drug–disease labels [2, 8], which can be limiting for newly studied phenotypes or for biobank settings where genotype and phenotype labels are available but disease-relevant molecular profiling is sparse or absent [2, 13–15]. Recent drug-repositioning and drug–target interaction prediction studies have increasingly used heterogeneous biological networks, graph representation learning, and multi-source biomedical knowledge integration to infer drug–target, drug–disease, and disease–mechanism relationships [16–19]. These approaches demonstrate the value of integrating chemical, biological, regulatory, and network-level information for drug repositioning and target-interaction prediction. Most such methods operate downstream of existing biomedical annotations and knowledge networks. In contrast, G2DR is designed for genotype-first settings, where individual-level genotype data and genetically predicted expression provide the initial disease signal before integration with pathway, network, druggability, and drug–target evidence. Thus, G2DR is complementary to recent DTI and heterogeneous-network-based drug-repositioning models rather than a direct replacement for them.

Human genetics provides a particularly attractive starting point for therapeutic prioritization because inherited variation is stable, scalable, and increasingly well characterized across large cohorts [13, 14]. When measured expression is unavailable, genotype can be propagated through transcriptome imputation models to estimate genetically regulated expression [20, 21], offering an interpretable intermediate layer between variant-level signal and candidate genes [15]. This strategy is appealing, but it also comes with an important caveats: TWAS-style approaches prioritize genes associated with trait-linked regulatory signal, not necessarily causal effector genes [15], and inference remains sensitive to linkage disequilibrium, co-regulation, tissue context, ancestry, and model architecture [15, 22]. These limitations argue for integrative frameworks that improve robustness and interpretability rather than relying on any single transcriptome-prediction resource or analytical test [15].

A further motivation comes from therapeutic translation. Multiple studies have shown that targets supported by human genetics are more likely to succeed in clinical development [23, 24], making genetics-informed target selection an increasingly important component of drug discovery. However, the key translational challenge is not simply to detect associated genes, but to prioritize those with the strongest convergent support and then connect them to tractable pharmacological hypotheses in a transparent and reproducible manner.

**Figure 1:**
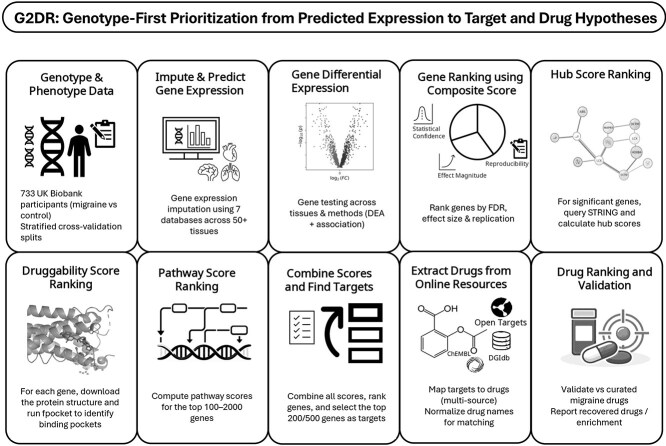
Overview of the G2DR framework. Genotype and phenotype data from the migraine cohort were partitioned by stratified cross-validation and propagated through genotype-based transcriptome imputation across multiple expression-weight resources. Covariate-adjusted predicted expression values were tested using multiple differential-expression and association methods, and significant results from the training and validation splits were aggregated into a discovery set. Genes were then ranked using a composite score that integrated reproducibility, effect magnitude, and statistical confidence, followed by pathway, network, and druggability annotation to generate an integrated target-prioritization score. Top-ranked genes were mapped to candidate compounds through Open Targets, DGIdb, and ChEMBL, and the resulting drug lists were evaluated against curated migraine-associated drug references.

Here, we present G2DR (Fig. 1), a genotype-first computational framework for genetics-informed target prioritization and drug repurposing hypothesis generation. G2DR integrates genetically predicted expression (Table 1) from multiple transcriptome-weight resources [15, 20, 21], multi-method gene-level testing, pathway enrichment, network context, structure- and knowledge-based druggability, and multi-source drug–target evidence [25–29] to generate ranked computational hypotheses for candidate targets and associated compounds, without requiring measured disease transcriptomics or supervised training on curated indication labels [2].

**Table 1:** Expression weight models used for genotype-based gene expression prediction.

Model	Source	Description	Gene–tissue pairs
MASHR	https://zenodo.org/records/7551845	Multivariate adaptive shrinkage models for cross-population transcriptome prediction, designed to improve TWAS performance in underrepresented populations [30]	686 241 (49 tissues)
JTI	https://zenodo.org/records/3842289	Joint-tissue imputation models that borrow information across tissues via shared genetic regulation, enabling both expression prediction and downstream causal inference [31]	533 141 (49 tissues)
CTIMP	https://github.com/yiminghu/CTIMP	Cross-tissue gene-expression imputation models trained with sparse group-LASSO to borrow information across GTEx tissues [32]	404 450 (49 tissues)
UTMOST	https://zenodo.org/records/3842289	Cross-tissue transcriptome prediction models from the UTMOST framework, hosted alongside JTI models; treated as a separate model set in this study [32]	340 104 (49 tissues)
EpiXcan	https://www.synapse.org/Synapse:syn52745052	Transcriptome imputation incorporating epigenomic annotations to weight SNP priors, improving prediction accuracy over standard elastic net [33]	143 609 (14 tissues)
FUSION	http://gusevlab.org/projects/fusion/	Bayesian sparse linear mixed model and penalized regression framework for transcriptome-wide association studies [21]	265 052 (48 tissues)
TIGAR	https://github.com/yanglab-emory/TIGAR	Nonparametric Bayesian gene expression imputation tool supporting individual- and summary-level GWAS data for transcriptome-wide association studies [34]	181 605 (49 tissues)

We demonstrate the framework in migraine using UK Biobank data and evaluate performance at multiple levels, including held-out gene replication, enrichment for curated migraine biology, and recovery of migraine-linked drugs from prioritized targets. Framed explicitly as a computational prioritization engine for downstream experimental follow-up rather than a clinically actionable target-identification system, G2DR is best viewed as a modular proof-of-concept framework with clear room for further maturation.

## Materials and methods

### Study design and cohort assembly

A total of 733 UK Biobank participants with available genotype data, migraine self-report status, and recorded comorbidity/covariate fields were included, comprising 53 migraine cases and 680 controls [13, 14]. Migraine status was based on the UK Biobank self-reported noncancer illness field. Recorded comorbidities and medication-related covariates were retained as cohort descriptors and potential sources of phenotypic heterogeneity, rather than as criteria defining a comorbidity-enriched migraine subtype. These comorbidities are commonly reported to co-occur with migraine [35–38]. Migraine status was based on the available UK Biobank self-reported non-cancer illness field; aura status, migraine subtype, specialist-confirmed diagnosis, and medication-supported case definitions were not available for reliable subtype-stratified analysis in the present cohort. Genotype–phenotype data were partitioned using stratified five-fold cross-validation, with each fold split into training (80%), validation (10%), and test (10%) subsets to preserve the case–control ratio. Genotype quality control was applied within each training fold and propagated to validation and test subsets, including per-variant filters for minor allele frequency $\geq 0.01$, Hardy–Weinberg equilibrium $P \geq 1\times 10^{-6}$, and variant missingness $\leq 0.1$, along with per-individual missingness $\leq 0.1$ and removal of related individuals using PLINK (--rel-cutoff 0.125) [39, 40].

### Genetically predicted gene expression

Genetically predicted gene expression was computed for each individual within each fold using PrediXcan-style genotype-based transcriptome imputation [20, 22], in which expression for gene $g$ in tissue $t$ is estimated as a weighted sum of SNP dosages using pre-trained expression-weight models. To maximize gene coverage and capture diverse regulatory architectures, we incorporated seven publicly available expression-weight databases trained under different statistical and biological assumptions: MASHR, JTI, CTIMP, UTMOST, EpiXcan, FUSION, and TIGAR (Table 1; full mathematical details are provided in Supplementary Methods S1). Predicted expression values were adjusted for sex and the Top-10 genetic principal components using ordinary least-squares regression applied to the training split only, with fitted parameters applied to validation and test splits.

### Differential expression analysis

Differential expression analysis was performed on covariate-adjusted imputed expression values for each gene, tissue, database, fold, and dataset split using eight statistical methods: LIMMA empirical Bayes moderated $t$-test [41], Welch’s unequal-variance $t$-test, OLS regression, Wilcoxon rank-sum test, phenotype-label permutation test ($B = 1{}000$ permutations), weighted logistic regression, bias-reduced Firth logistic regression, and Bayesian logistic approximation. For differential-expression methods, significance required FDR $< 0.1$ and $|\log _{2}\mathrm{FC}| \geq 0.5$; for association-style methods, significance required false discovery rate, FDR $< 0.1$ and $|\mathrm{Effect}| \geq 0.5$. Complete model specifications are provided in Supplementary Methods S1.

### Gene prioritization

Significant genetically predicted expression-associated genes were aggregated across all databases, tissues, folds, and methods. The discovery set was defined as $G_{\mathrm{discovery}} = G_{\mathrm{train}} \cup G_{\mathrm{val}}$, reserving $G_{\mathrm{test}}$ exclusively for internal held-out evaluation. Each gene in $G_{\mathrm{discovery}}$ was scored using a primary predicted-expression evidence score, denoted $\mathrm{DEbase}_{g}$, that integrates three components: reproducibility (40%), effect magnitude (30%), and statistical confidence (30%). Reproducibility captures both the total number of significant hits and the breadth of support across database–tissue–method combinations. Effect magnitude is derived from method-standardized absolute effect sizes. Statistical confidence is derived from BH-adjusted FDR values. Full scoring formulas are provided in Supplementary Methods S2. Rankings were robust to alternative weights (Spearman $\rho = 0.992$; Top-100 overlap $\geq 75\%$; full stability results in Supplementary Table S1).

Internal held-out replication was assessed using the held-out test split from the same UK Biobank-derived cohort as the replication target. For each gene $g$ in the tested universe $U$, a binary label $y_{g} = \mathbb{I}(g \in G_{\mathrm{test}})$ was defined, and $\mathrm{DEbase}_{g}$ was used as the ranking predictor. Performance was quantified using Receiver Operating Characteristic Area Under the Curve (ROC–AUC), Area Under the Precision-Recall Curve (PR-AUC), and hypergeometric enrichment tests at multiple Top-$K$ cutoffs. Enrichment for a curated set of 3190 migraine-associated genes [15] assembled via PhenotypeToGeneDownloaderR (https://github.com/MuhammadMuneeb007/PhenotypeToGeneDownloaderR) was assessed using the same hypergeometric framework. Full test derivations are provided in Supplementary Methods S3. To address potential opportunity bias introduced by aggregating significance across multiple databases, tissues, methods, and folds, we additionally performed opportunity-corrected and stricter aggregation sensitivity analyses. The opportunity-corrected analysis used the per-gene hit rate, defined as the number of significant discovery hits divided by the number of valid discovery testing opportunities for that gene. Stricter aggregation analyses required recurrent support across folds, databases, tissues, methods, database–tissue–method contexts, and replicated direction of effect. These analyses are described in Supplementary Methods S6 and reported in Supplementary Tables S11 and S12. Because the primary analysis used all available expression-weight tissues, we additionally performed a tissue-group sensitivity analysis to evaluate whether migraine-relevant tissue subsets retained enrichment and internal replication signal. Tissues were grouped into all tissues, brain/CNS, vascular/cardiovascular, and immune/blood categories, and Top-$K$ enrichment for curated migraine genes and held-out test-positive genes was evaluated within each group. This analysis is described in Supplementary Methods S7 and reported in Supplementary Table S13.

To obtain an integrated target-prioritization ranking, the primary predicted-expression evidence score $\mathrm{DEbase}_{g}$ was combined with three additional evidence layers: pathway support (*PathwayScore*) derived from GO, KEGG, Reactome, and Disease Ontology enrichment analyses (clusterProfiler, ReactomePA, DOSE); network hub score from STRING protein–protein interaction analysis; and structure- or knowledge-based druggability from DGIdb, ChEMBL, and fpocket. The integrated score was computed as $\mathrm{CoreScore}_{g} = 0.45 \cdot \mathrm{DE}^{\mathrm{norm}}_{g} + 0.25 \cdot \mathrm{Path}^{\mathrm{norm}}_{g} + 0.25 \cdot \mathrm{Drug}^{\mathrm{norm}}_{g} + 0.05 \cdot \mathrm{Hub}^{\mathrm{norm}}_{g}$, with DE weighted highest because it provides the primary TWAS-derived genetic link between genotype and disease. Here, $\mathrm{DE}^{\mathrm{norm}}_{g}$ denotes the normalized form of $\mathrm{DEbase}_{g}$, the primary predicted-expression evidence score derived from reproducibility, effect magnitude, and statistical confidence. It is not a separate measured transcriptomic differential-expression component. Full pathway score propagation formulas and weight justification are provided in Supplementary Methods S2. The notation used in this scoring framework is summarized in Table 2.

**Table 2:** Notation used to distinguish the SNP set for expression prediction, the primary predicted-expression evidence score, normalized evidence layers, and the final integrated gene-prioritization score.

Term	Meaning
$\mathcal{V}_{g,t}$	SNPs used to predict genetically regulated expression of gene $g$ in tissue $t$
$\mathrm{DEbase}_{g}$	Primary gene-level predicted-expression evidence score derived from reproducibility, effect magnitude, and statistical confidence
$\mathrm{DE}^{\mathrm{norm}}_{g}$	Normalized form of $\mathrm{DEbase}_{g}$ used in the integrated score; not a separate measured transcriptomic differential-expression component
$\mathrm{Path}^{\mathrm{norm}}_{g}$	Normalized pathway-support component used in $\mathrm{CoreScore}_{g}$
$\mathrm{Drug}^{\mathrm{norm}}_{g}$	Normalized druggability or drug–target evidence component used in $\mathrm{CoreScore}_{g}$
$\mathrm{Hub}^{\mathrm{norm}}_{g}$	Normalized network hub component used in $\mathrm{CoreScore}_{g}$
$\mathrm{CoreScore}_{g}$	Final integrated target-prioritization score combining predicted-expression evidence, pathway support, druggability or drug–target evidence, and network hub support

### Drug repurposing

A curated migraine drug reference set of 4824 normalized drug entries was assembled using DownloadDrugsRelatedToDiseases (https://github.com/MuhammadMuneeb007/DownloadDrugsRelatedToDiseases), which aggregates drug–disease associations from Open Targets, DrugBank, CTD, and literature-mining sources. All enrichment claims are framed relative to a global background of 139 597 drugs. Candidate compounds were mapped from the Top-$N$ ranked genes using drug–target evidence from Open Targets, DGIdb, and ChEMBL, with drug names harmonized using conservative text normalization. Each gene–drug evidence row was assigned an evidence weight based on clinical development stage, and a *DrugScore* was computed by aggregating $\mathrm{GeneWeight} \times \mathrm{EvidenceWeight}$ contributions across all supporting genes.

The curated migraine drug reference set was partitioned into four development-stage tiers: Tier 1 (migraine-specific approved therapies), Tier 2 (guideline-supported acute or preventive therapies), Tier 3 (established off-label therapies), and Tier 4 (broader literature-linked compounds). Performance was assessed using multi-$K$ overlap evaluation under two complementary universes: an ALL-drugs global background (hypergeometric test valid) and a PREDICTED candidates-only universe (AUROC and AUPRC are the appropriate metrics; full results in Supplementary Table S5).

### Directionality assessment

To assess mechanistic compatibility of prioritized gene–drug pairs, drug action annotations were extracted from locally aggregated evidence fields and supplemented via ChEMBL and DGIdb, harmonized into a reduced action vocabulary (inhibitor, antagonist, agonist, activator, modulator, and unknown). Each pair was classified as directionally consistent when the drug action was compatible with the inferred disease-associated gene direction, inconsistent when opposed, or unclear when insufficient annotation was available. Full annotation and classification criteria are provided in Supplementary Methods S4.

## Results

### Cross-database concordance of predicted gene expression

A total of 733 participants (53 cases, 680 controls) were stratified into five cross-validation folds, preserving case–control balance across training (80%), validation (10%), and test (10%) splits. Within each fold and split, we imputed genetically regulated expression from genotype dosages using PrediXcan-style models [20, 22].

**Figure 2:**
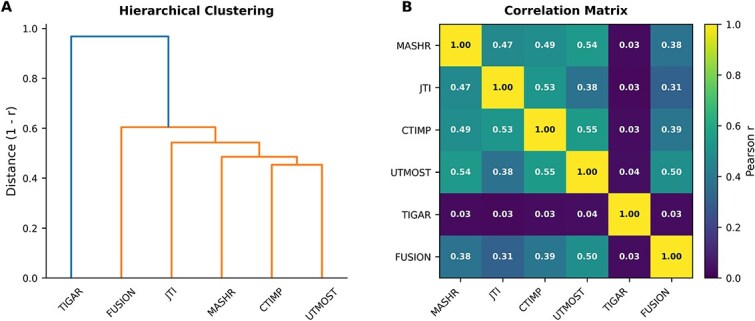
Cross-database concordance of predicted gene expression across expression-weight databases. (A) Hierarchical clustering of databases using distance $(1-r)$ derived from the pairwise correlation matrix. (B) Pairwise correlation matrix (Pearson $r$).

To compare expression-weight databases, we quantified tissue-matched concordance by computing gene-wise Pearson correlations of predicted expression across individuals for genes shared by each database pair, then averaging within tissue and across folds to yield one concordance estimate per database pair and split. The highest concordance was observed for the CTIMP–UTMOST pair ($r \approx 0.55$; train $= 0.547$, validation $= 0.546$, test $= 0.546$), followed by JTI–CTIMP ($r \approx 0.53$; train $= 0.526$, validation $= 0.525$, test $= 0.525$), MASHR–UTMOST ($r \approx 0.54$; train $= 0.536$, validation $= 0.538$, test $= 0.537$), and MASHR–CTIMP ($r \approx 0.49$; train $= 0.491$, validation $= 0.492$, test $= 0.492$). Concordance with FUSION was moderate for UTMOST ($r \approx 0.50$; train $= 0.498$, validation $= 0.497$, test $= 0.499$) and lower for MASHR ($r \approx 0.38$; train $= 0.380$, validation $= 0.380$, test $= 0.378$) and JTI ($r \approx 0.31$; train $= 0.311$, validation $= 0.310$, test $= 0.311$). TIGAR showed near-zero concordance with all other databases ($r \approx 0.03$), indicating substantially different predicted-expression patterns (Fig. 2).

Adjusting expression for sex and the Top-10 genetic principal components had negligible impact on cross-database correlations (overall mean $\Delta r = r_{\mathrm{fixed}} - r_{\mathrm{raw}} \approx -1 \times 10^{-4}$). However, the adjustment altered expression values modestly overall ($\mathrm{corr}(\mathrm{raw},\mathrm{fixed}) = 0.9696$, mean$|\Delta | = 0.00797$, RMSE $= 0.0353$), with the largest shifts observed in FUSION (mean$|\Delta | \approx 0.023$, RMSE $\approx 0.124$) and in EpiXcan (mean$|\Delta | \approx 0.011$, RMSE $\approx 0.078$), while TIGAR showed minimal absolute change (mean$|\Delta | \approx 6.3 \times 10^{-5}$). These results suggest that cross-database differences primarily reflect methodological and architectural variation among expression-weight resources, rather than confounding by sex or ancestry principal components.

### Gene differential expression analysis

We performed 172 868 680 gene-level differential-expression and association tests across expression-weight databases, tissues, folds, dataset splits, and eight statistical methods. Applying the predefined significance and effect-size thresholds yielded 96 502 significant test records, corresponding to 11 451 unique significant genes within a tested universe of $U = 34{\,}355$ genes. Given the modest cohort size and the highly layered testing design, these counts should be interpreted primarily as a broad discovery space rather than as a set of individually robust causal signals. Importantly, the main ranking conclusions were preserved under stricter thresholds in sensitivity analyses, indicating that the held-out prioritization signal does not depend solely on the most permissive operating point. Discovery candidates were defined without test leakage using training and validation only, producing $G_{\mathrm{discovery}} = G_{\mathrm{train}} \cup G_{\mathrm{val}}$ ($n = 9{}305$), of which 1189 overlapped a curated migraine-linked reference set and 8116 remained outside that reference space.

Internal held-out replication was assessed by treating test-significant genes ($T = 7{}141$) from the same UK Biobank-derived analytical framework as positives within $U$, where $G_{\mathrm{discovery}}$ recovered 4995 test positives (recall $= 0.699$) with precision $= 0.537$, while smaller Top-$K$ lists achieved higher precision at the cost of recall (e.g. Top-100 precision $= 0.790$; Table 3).

**Table 3:** Held-out test replication for the discovery set and Top-$K$ lists, with positives test-significant genes ($T = 7{}141$), and TP/FP/FN computed relative to $T$.

Predicted set	TP	FP	FN	Precision	Recall
$G_{\mathrm{discovery}}$ (train $\cup$ val)	4995	4310	2146	0.537	0.699
Top 50	39	11	7102	0.780	0.005
Top 100	79	21	7062	0.790	0.011
Top 200	156	44	6985	0.780	0.022
Top 500	347	153	6794	0.694	0.049
Top 1000	664	336	6477	0.664	0.093

We next evaluated the continuous discovery score as a genome-wide ranking model over the full tested universe $U$, using test-significant genes ($T = 7{}141$) as the replication target. Under this ranking framework, the score achieved ROC–AUC $= 0.7753$ and PR-AUC $= 0.4754$, indicating meaningful separation of test-replicating genes from nonreplicating genes. Given the baseline prevalence of positives in the tested universe ($T/U = 0.2079$), the observed PR-AUC corresponds to a substantial lift over random ranking. We therefore interpret the primary signal of G2DR at this stage not as binary hit identification, but as the ability to order genes such that held-out positives are preferentially concentrated toward the top of the ranked list. This distinction is important in small, class-imbalanced settings, where prioritization quality is more informative than exact gene-list identity (Table 4, left panel).

**Table 4:** Hypergeometric enrichment of predicted sets for held-out test positives and curated migraine genes, with test positives: $k_{\mathrm{exp}} = N_{\mathrm{pred}} \cdot (T/U)$, $T = 7{}141$, $U = 34{\,}355$ migraine genes: $k_{\mathrm{exp}} = N_{\mathrm{pred}} \cdot (|M|/U)$, and $|M \cap U| = 3{}190$ FE = $k_{\mathrm{obs}}/k_{\mathrm{exp}}$.

	Test positives ($T = 7{}141$)					Curated migraine genes ($|M| = 3{}190$)				
Set	$N_{\mathrm{pred}}$	$k_{\mathrm{obs}}$	$k_{\mathrm{exp}}$	FE	$P$ -value	$N_{\mathrm{pred}}$	$k_{\mathrm{obs}}$	$k_{\mathrm{exp}}$	FE	$P$ -value
$G_{\mathrm{discovery}}$ (train $\cup$ val)	9305	4995	1934.13	2.58	$<10^{-300}$	9305	1189	864.01	1.38	$5.47\times 10^{-40}$
Top 50	50	39	10.39	3.75	$7.14\times 10^{-18}$	50	12	4.64	2.58	$1.71\times 10^{-3}$
Top 100	100	79	20.79	3.80	$1.55\times 10^{-35}$	100	21	9.29	2.26	$2.97\times 10^{-4}$
Top 200	200	156	41.57	3.75	$1.77\times 10^{-67}$	200	36	18.57	1.94	$8.72\times 10^{-5}$
Top 500	500	347	103.93	3.34	$6.94\times 10^{-123}$	500	74	46.43	1.59	$4.26\times 10^{-5}$
Top 1000	1000	664	207.86	3.19	$2.30\times 10^{-220}$	1000	132	92.85	1.42	$2.39\times 10^{-5}$

As an orthogonal validation, we tested enrichment for curated migraine genes ($M = 3{}190$) within $U$. $G_{\mathrm{discovery}}$ contained 1189 known genes (expected 864.01), corresponding to 1.38-fold enrichment ($P = 5.47 \times 10^{-40}$), indicating that the discovery set preferentially captures established migraine biology while retaining a large novel component (Table 4, right panel).

To quantify cross-fold reproducibility, we performed a fold-specific stability analysis in which training and validation records were combined within each fold and genes were ranked using fold-local predicted-expression evidence. Pairwise Jaccard similarity across Top-$K$ gene lists was modest, ranging from 0.0618 at Top-100 to 0.0929 at Top-1000, but consistently exceeded random Top-$K$ baselines (0.0007–0.0148). Genes shared by all five folds increased from 2 at Top-50 to 22 at Top-1000, and fold-specific Top-$K$ unions were enriched for curated migraine genes. These results indicate modest but nonrandom cross-fold stability, with full results provided in Supplementary Table S8.

Together, these results show that integrating gene-level evidence across tissues, methods, and databases yields reproducible internal held-out prioritization within the same UK Biobank-derived analytical framework, performs substantially better than random ranking, and shows modest but above-random cross-fold stability. These findings support the use of G2DR as a prioritization framework for generating compact candidate gene sets for downstream network and drug-mapping analyses, while reinforcing that individual gene-level rankings should be interpreted cautiously rather than as perfectly fold-invariant targets.

### Gene prioritization was robust to stricter significance thresholds

To assess whether the discovery-based prioritization was sensitive to the choice of significance threshold, we repeated the same workflow under stricter FDR and effect-size criteria. Relative to the primary analysis (FDR $< 0.10$, $|\log _{2}\mathrm{FC}| \geq 0.50$), tightening the FDR threshold to $< 0.05$ reduced the number of significant genes in training, validation, and test from 1046, 9107, and 7141 to 958, 7861, and 5313, respectively, and reduced $G_{\mathrm{discovery}}$ from 9305 to 8135 genes. Increasing the effect-size threshold to $|\log _{2}\mathrm{FC}| \geq 0.75$ at FDR $< 0.10$ reduced these counts further to 263, 6241, and 4599 genes, yielding $G_{\mathrm{discovery}} = 6{}303$, while the strictest setting (FDR $< 0.05$, $|\log _{2}\mathrm{FC}| \geq 0.75$) produced 248 training genes, 5709 validation genes, and 3772 test genes, with $G_{\mathrm{discovery}} = 5{}794$.

Despite the expected reduction in $G_{\mathrm{discovery}}$ size under stricter filtering, held-out replication remained substantial across all threshold settings. The primary analysis achieved ROC–AUC $= 0.7753$ and PR-AUC $= 0.4754$. Under FDR $< 0.05$ and $|\log _{2}\mathrm{FC}| \geq 0.50$, performance remained above random with ROC–AUC $= 0.7314$ and PR-AUC $= 0.3412$. Under the stricter effect-size threshold of $|\log _{2}\mathrm{FC}| \geq 0.75$, performance was similarly retained, with ROC–AUC $= 0.7339$ and PR-AUC $= 0.3289$ for FDR $< 0.10$, and ROC–AUC $= 0.7025$ and PR-AUC $= 0.2497$ for FDR $< 0.05$. $G_{\mathrm{discovery}}$ also remained strongly enriched for held-out test positives across all settings, with fold enrichment ranging from 2.58-fold to 3.20-fold. Enrichment for curated migraine genes was likewise preserved, ranging from 1.38-fold under the primary rule to 1.41-fold under the strictest setting. Full sensitivity results are provided in Supplementary Table S2; together, these results indicate that the main gene-prioritization findings are not driven by permissive significance filtering.

### Component-based recovery

To identify which components contributed the most disease-relevant signal in $G_{\mathrm{discovery}}$, we tested whether each component-specific discovery set (defined using training and validation only) was enriched for curated migraine-associated genes. For each database, tissue, and method, we defined a discovery set as the unique genes that were significant at least once within that component across folds and the remaining dimensions, and quantified over-representation of database-annotated migraine-linked genes using a fold-enrichment statistic (FE $= k_{\mathrm{obs}}/k_{\mathrm{exp}}$) with empirical $P$-values from size-matched random gene-set sampling. Full results across all databases, tissues, and methods are provided in Supplementary Table S3. The primary analysis used all available expression-weight tissues because G2DR is intended for genotype-first settings in which the disease-relevant tissue may be uncertain, inaccessible, or unavailable in reference transcriptomic resources. This all-tissue strategy maximized gene coverage and avoided excluding regulatory signals *a priori*, but it was not intended to imply that all tissues are equally relevant to migraine. This is important because migraine involves neurological, trigeminovascular, vascular, immune/inflammatory, endocrine, and systemic mechanisms, while trigeminal ganglion, a highly relevant migraine tissue, was not available among the GTEx-derived expression-weight resources used here. Consistent with this rationale, the component-level enrichment analysis in Supplementary Table S3 identified biologically plausible tissue contexts among the top enriched tissues, including Brain Amygdala, Brain Anterior Cingulate Cortex BA24, Brain Spinal Cord Cervical C-1, Whole Blood, Cells EBV-transformed Lymphocytes, and Artery Coronary. Nevertheless, inclusion of many tissues may introduce noise or dilute tissue-specific signals; therefore, tissue-level findings should be interpreted as broad genetically regulated expression evidence rather than definitive tissue-specific migraine mechanisms. To further assess whether the all-tissue strategy was dominated by potentially unrelated tissues, we performed a tissue-group sensitivity analysis comparing all tissues with brain/CNS, vascular/cardiovascular, and immune/blood subsets. Tissue-prioritized groups retained internal held-out replication signal above random expectation. At Top-100, test-positive gene enrichment was observed for all tissues (FE = 2.97, $P = 4.90\times 10^{-19}$), brain/CNS tissues (FE = 6.69, $P = 3.82\times 10^{-42}$), vascular/cardiovascular tissues (FE = 11.37, $P = 9.86\times 10^{-49}$), and immune/blood tissues (FE = 14.19, $P = 5.00\times 10^{-50}$). Curated migraine-gene enrichment was also retained in the all-tissue, brain/CNS, and immune/blood groups, while the vascular/cardiovascular group showed stronger internal replication than curated-gene enrichment. These findings support the use of an all-tissue strategy for broad genotype-first discovery while indicating that migraine-relevant tissue subsets contribute meaningful signal. Full tissue-group sensitivity results are provided in Supplementary Table S13.

Across expression-weight databases, MASHR showed the strongest discovery enrichment (FE $= 2.30$, $P_{\mathrm{emp}} = 1\times 10^{-4}$), followed by JTI (FE $= 1.86$, $P_{\mathrm{emp}} = 6.799\times 10^{-3}$) and FUSION (FE $= 1.36$, $P_{\mathrm{emp}} = 1\times 10^{-4}$), whereas EpiXcan, CTIMP, UTMOST, and TIGAR showed weaker and nonsignificant enrichment. Across tissues, discovery enrichment highlighted a reproducible set of migraine-informative contexts, led by Brain Amygdala (FE $= 1.96$, $P_{\mathrm{emp}} = 1\times 10^{-4}$), Minor Salivary Gland (FE $= 1.94$, $P_{\mathrm{emp}} = 1\times 10^{-4}$), and Whole Blood (FE $= 1.83$, $P_{\mathrm{emp}} = 1\times 10^{-4}$), with multiple additional tissues showing significant over-representation of database-annotated migraine-linked genes. Across methods, the discovery signal was dominated by the association-style models: Weighted Logistic and Bayesian Logistic both showed consistent enrichment (FE $\approx 1.37$, $P_{\mathrm{emp}} = 1\times 10^{-4}$), while Welch $t$-test identified a smaller, disease-dense set (FE $= 1.35$, $P_{\mathrm{emp}} = 6\times 10^{-4}$); low-coverage methods did not show significant discovery enrichment.

### Unified gene prioritization framework

We next evaluated a unified gene-prioritization framework by comparing individual evidence components against both the primary composite score and the multi-source integrated score. Two complementary evaluation universes were used throughout. The **full universe** comprised all $U = 34{\,}355$ tested genes; genes not in the discovery set received a score of zero, so this universe measures how well a ranking separates discovery-relevant genes from the entire tested space—the same universe used to compute the headline ROC–AUC and PR-AUC reported above. The **discovery universe** comprised only the $n = 9{}305$ genes in $G_{\mathrm{discovery}}$ (train $\cup$ val); here nondiscovery genes are absent, so this universe measures ranking quality *within* the already-selected candidates—a strictly harder question.

Two composite scores were evaluated. The **primary predicted-expression evidence score** ($\mathrm{DEbase}_{g}$, *Importance:Score*) integrates reproducibility, effect magnitude, and statistical confidence with weights 40/30/30. The **integrated score** additionally combines $\mathrm{DEbase}_{g}$ with pathway support, druggability evidence, and network hub score (weights DE $= 0.45$, Pathway $= 0.25$, Drug $= 0.25$, Hub $= 0.05$), and is designed for downstream target selection.

Evaluated over all 34 355 tested genes, effect-only ranking achieved the strongest test-replication performance (ROC–AUC $= 0.790$, PR-AUC $= 0.526$), marginally exceeding the primary composite (ROC–AUC $= 0.775$, PR-AUC $= 0.475$) and the integrated score (ROC–AUC $= 0.776$, PR-AUC $= 0.472$). The elevated AUC values across most rankings in this universe reflect the large separation between discovery genes (score $> 0$) and the majority of nondiscovery genes (score $= 0$), rather than solely fine-grained within-set discrimination. For curated migraine-gene enrichment at Top-200, pathway ranking recovered 66 known genes (FE $= 3.55$, precision $= 0.330$) and hub ranking recovered 64 (FE $= 3.45$, precision $= 0.320$), both substantially exceeding the primary composite (36 genes; FE $= 1.94$) and significance-only (25 genes; FE $= 1.35$) rankings.

Within $G_{\mathrm{discovery}}$ alone, effect-only ranking remained the strongest for test replication (ROC–AUC $= 0.675$, PR-AUC $= 0.663$), recovering 145 held-out test-positive genes at Top-200. The primary composite and significance-only rankings showed similar within-set replication (ROC–AUC $\approx 0.54$–$0.55$), indicating that the headline 0.775 figure is driven in substantial part by discovery–versus–nondiscovery separation rather than by within-set ranking quality alone. Pathway and hub rankings showed substantially stronger enrichment for curated migraine biology: at Top-200, pathway ranking recovered 66 database-annotated migraine-linked genes (FE $= 2.58$, precision $= 0.330$) and hub ranking recovered 64 (FE $= 2.50$, precision $= 0.320$). Direct target evidence recovered 58 known genes (FE $= 2.27$, precision $= 0.290$), whereas drug-link count and druggability alone provided weaker standalone biological enrichment.

Component-level benchmarking showed that no single evidence layer was uniformly optimal across all evaluation criteria. Effect-only ranking performed best for held-out replication, whereas pathway- and hub-based rankings were stronger for enrichment of curated migraine biology. The integrated score therefore should not be interpreted as maximizing any single benchmark metric; rather, it represents a balanced operating point designed for downstream target selection, where replication support, biological coherence, and translational tractability all matter simultaneously. In this sense, the value of integration lies in controlled trade-off rather than empirical dominance on one axis alone. Full component-wise metrics across both universes are provided in Supplementary Table S4. This distinction was important for interpreting the ranking metrics. The original $\mathrm{DEbase}_{g}$ score achieved full-universe test ROC–AUC $= 0.7753$ and PR-AUC $= 0.4754$, but within $G_{\mathrm{discovery}}$ the corresponding test ROC–AUC was more modest ($0.5431$). Thus, the full-universe ROC–AUC primarily reflects separation between discovery-supported genes and genes without discovery evidence, rather than strong fine-grained discrimination among already-selected candidates. To address possible opportunity bias from genes tested across more databases, tissues, methods, or folds, we repeated the analysis using an opportunity-corrected score and stricter aggregation rules. The opportunity-corrected $\mathrm{DEbase}^{\mathrm{opp}}_{g}$ retained full-universe performance (ROC–AUC $= 0.7779$, PR-AUC $= 0.4767$) and modestly improved within-discovery test ROC–AUC to $0.5660$. Stricter recurrence rules reduced the number of nonzero-scored genes, as expected, but several retained internal replication and Top-$K$ enrichment signals. These results support a cautious interpretation: G2DR provides evidence of enrichment and internal replication, while fine-grained prioritization within the selected discovery set remains more modest. Full results are provided in Supplementary Tables S11 and S12.

To assess whether the integrated score weights were arbitrary, we evaluated 17 alternative weighting schemes spanning nine reasonable alternatives (e.g. DE-heavy, pathway-heavy, drug-heavy, equal, and no-hub variants) and eight extreme stress tests (single-component schemes). Across reasonable alternatives, the mean Spearman $\rho$ against the default ranking was $0.963$ (minimum $0.866$), with a mean Top-100 gene overlap of $81.6\%$; crucially, performance metrics were virtually unchanged, with DISC-universe test ROC–AUC ranging only from $0.544$ to $0.547$ and FULL-universe test ROC–AUC from $0.775$ to $0.776$ across all nine schemes (Supplementary Table S1). Extreme single-component schemes diverged substantially (mean $\rho = 0.682$, minimum $0.292$ for hub-only), suggesting that the integrated score draws on genuine multi-source signal rather than being dominated by any single layer. These results suggest that the default weights (DE $= 0.45$, Pathway $= 0.25$, Drug $= 0.25$, Hub $= 0.05$) lie within a stable region of parameter space, with divergence occurring only under extreme parameterizations that lack biological justification in a TWAS-first pipeline.

### Significant gene comparison against open targets

We compared G2DR with the Open Targets migraine disease–target resource using the same curated migraine reference set ($M = 3{}190$ genes). In the full analysis, G2DR ranked 9305 genes across the broader genotype-first search space, whereas Open Targets returned 2376 migraine-associated targets. The full G2DR ranking recovered 1189 curated migraine genes (37.27%), while Open Targets recovered 1228 (38.50%), and G2DR additionally recovered 725 curated migraine reference genes absent from Open Targets entirely. When G2DR was restricted to genes represented in the Open Targets target space ($n = 823$), Top-50 precision increased from 22.00% to 46.00% and Top-200 precision increased from 23.00% to 55.50%, approaching but not matching the standalone Open Targets precision (Top-50: 92.00%), which reflects its disease-curated, prefiltered design rather than a direct performance comparison. Full comparison metrics are provided in Supplementary Table S6. These comparisons are complementary rather than directly competitive: Open Targets offers higher precision within a curated space, while G2DR extends target hypothesis generation beyond existing disease annotations, recovering a substantial set of curated migraine reference genes that are absent from Open Targets entirely.

### Drug mapping and candidate enrichment

Using the ranked migraine gene lists (top $N = 200$ and $N = 500$), we aggregated gene–drug evidence from Open Targets, DGIdb, and ChEMBL to generate ranked candidate compounds. For $N = 200$, the pipeline compiled 5348 gene–drug evidence rows and yielded 3963 unique predicted drugs. For $N = 500$, evidence increased to 11 234 rows and the predicted set expanded to 7981 unique drugs.

We evaluated overlap with a curated reference set of 4824 normalized migraine-associated drugs under two complementary evaluation frames. First, an **ALL-drugs universe** (139 597 background drugs) tests capture and enrichment: whether the returned candidates include migraine-linked drugs above chance relative to the global pharmacopeia. Second, a **PREDICTED universe** (3963 or 7981 returned drugs) evaluates ranking within the returned candidate set: whether reference migraine-associated drugs are preferentially placed toward the top among returned compounds. Note that FE values below 1.0 in the PREDICTED universe reflect the fact that the reference migraine drug set exceeds the returned candidate pool in size, making standard hypergeometric enrichment interpretation inapplicable in that frame; AUROC and AUPRC are the appropriate metrics for within-set ranking performance.

Under the ALL-drugs background, both gene sets showed enrichment for migraine-linked compounds relative to a global drug background. For $N = 200$, recovery increased from $5/20$ (Precision@20 $= 0.25$, FE $= 7.23$, $P = 4.93\times 10^{-4}$) to $51/100$ (Precision@100 $= 0.51$, FE $= 14.76$, $P = 4.20\times 10^{-47}$) and $205/500$ (Precision@500 $= 0.41$, FE $= 11.86$, $P = 6.19\times 10^{-161}$). For $N = 500$, early enrichment improved further ($8/20$, Precision@20 $= 0.40$, FE $= 11.58$, $P = 1.75\times 10^{-7}$; $53/100$, Precision@100 $= 0.53$, FE $= 15.34$, $P = 4.48\times 10^{-50}$; $213/500$, Precision@500 $= 0.426$, FE $= 12.33$, $P = 1.67\times 10^{-171}$). These results indicate that both gene inputs capture broad migraine-relevant pharmacological signal relative to a global drug background, but they do not by themselves demonstrate recovery of migraine-specific approved therapeutic classes.

Within the returned candidates, for $N = 200$, 353 curated migraine drugs were present among 3963 predicted drugs and the ranking achieved AUROC $= 0.8004$ and AUPRC $= 0.3528$. For $N = 500$, 527 curated migraine drugs were present among 7981 predicted drugs with AUROC $= 0.8152$ and AUPRC $= 0.3311$. Expanding the input from 200 to 500 genes increased the number of recovered reference drugs and modestly improved AUROC, while AUPRC decreased slightly due to the larger candidate universe. Together, the two-universe evaluation separates capture from ranking: fold-enrichment and hypergeometric significance under the ALL universe support broad disease-relevant pharmacological signal in the upstream gene prioritization, while AUROC and AUPRC suggest that reference migraine-associated drugs tend to appear earlier than nonreference drugs within the returned set. These metrics should be interpreted as evidence of knowledge-base concordance and pharmacological hypothesis generation, not as validation that the framework recovers migraine-specific approved therapies. Full multi-$K$ overlap results for both $N = 200$ and $N = 500$ under both universes are provided in Supplementary Table S5. Because candidate drug generation and migraine-reference construction can draw on overlapping biomedical resources, we additionally performed a source-overlap sensitivity analysis to distinguish knowledge-base concordance from source-disjoint recovery; full source distributions and sensitivity results are provided in Supplementary Tables S9 and S10. Importantly, this signal was not explained solely by shared prediction/reference sources: after excluding reference drugs supported by Open Targets, DGIdb, or ChEMBL, the source-disjoint reference set still showed substantial enrichment among ranked predictions, including 43/100 Top-100 drugs (FE = 13.54, $P = 1.98 \times 10^{-37}$) and 78/200 Top-200 drugs (FE = 12.28, $P = 1.10 \times 10^{-62}$).

When we mapped the predicted drug set back to disease indications in the aggregated drug–disease database, migraine was not always the top recovered indication. Instead, cardiometabolic, inflammatory, neuropsychiatric, and seizure-related categories frequently ranked highly. This is biologically plausible because migraine has a well-established comorbidity landscape and shares mechanisms, risk factors, and therapeutic overlap with multiple neurological and systemic disorders [35–38]. Disease-label recovery should therefore be interpreted as reflecting shared pharmacology and overlapping biology across comorbid conditions rather than as a migraine-exclusive signal. We partitioned the curated migraine drug reference set ($n = 4{}824$ normalized drugs) into four evidence-oriented tiers: migraine-specific approved therapies (Tier 1; triptans, ditans, gepants, CGRP monoclonals, ergot derivatives), guideline-supported acute or preventive therapies (Tier 2), established off-label therapies (Tier 3), and broader literature-linked compounds (Tier 4). Recovery of each tier was evaluated within the ranked candidate drug list against a global background of 139 597 drugs.

Across the pooled migraine-drug reference set, the ranked list recovered migraine-linked compounds at rates above random expectation, but tiered evaluation showed that this recovery was uneven across evidence classes. No Tier 1 migraine-specific approved therapies were recovered within the Top-200 ranked drugs, whereas overlap was more evident for guideline-supported, off-label, and broader literature-linked compounds. The strongest and most statistically robust signal was observed for Tier 4 broader literature-linked compounds, with limited recovery of migraine-specific approved therapeutic classes. These results indicate that the current framework is better suited to broad pharmacological hypothesis generation than to rediscovering modern migraine-specific therapeutic classes. We therefore interpret the drug-level signal as evidence of broad mechanism-linked and repurposing-relevant pharmacology rather than indication-specific clinical precision (Table 5).

**Table 5:** Tiered migraine-evidence benchmark of the ranked drug list, with Tier 1: migraine-specific approved therapies ($n_{\mathrm{ref}}=135$); Tier 2: guideline-supported therapies ($n_{\mathrm{ref}}=92$); Tier 3: established off-label therapies ($n_{\mathrm{ref}}=29$); Tier 4: broader literature-linked compounds ($n_{\mathrm{ref}}=4{}568$), Fold enrichment (FE) and hypergeometric $P$-values are computed relative to a global background of 139 597 drugs but high FE values for Tiers 2–3 reflect low absolute counts and should be interpreted accordingly.

Tier	Top-20	Top-50	Top-100	Prec@100	Rec@100	FE@100	$P$ @100	Top-200
Tier 1: Migraine-specific approved	0	0	0	0.00	0.000	0.00	1.00	0
Tier 2: Guideline-supported	0	1	2	0.02	0.022	30.35	$2.04\times 10^{-3}$	2
Tier 3: Established off-label	1	1	1	0.01	0.034	48.14	$2.06\times 10^{-2}$	1
Tier 4: Broad literature-linked	4	18	48	0.48	0.011	14.67	$7.08\times 10^{-44}$	86

### Directionality of prioritized gene–drug pairs

To assess mechanistic plausibility beyond drug enrichment alone, we evaluated the directionality of all gene–drug pairs derived from the Top-200 genes of the final integrated migraine ranking. Across $4{}861$ unique gene–drug pairs, 549 (11.3%) were classified as directionally consistent, 618 (12.7%) as inconsistent, and $3{}694$ (76.0%) as unclear (Table 6). When restricted to the 614 pairs involving drugs from the curated migraine reference set, 50 (8.1%) were consistent, 83 (13.5%) inconsistent, and 481 (78.3%) unclear. The proportions were broadly stable across both universes, indicating that the directionality signal does not selectively concentrate in migraine-annotated compounds.

**Table 6:** Summary of directionality classification for unique prioritized gene–drug pairs from the final integrated migraine ranking, with results shown for all pairs derived from the Top-200 ranked genes and separately for pairs involving drugs from the curated migraine reference set ($n_{\mathrm{mig}}=614$).

Directionality class	All pairs ($n=4{}861$)		Migraine drugs only ($n=614$)	
	Count	%	Count	%
Consistent	549	11.3	50	8.1
Inconsistent	618	12.7	83	13.5
Unclear	$3{}694$	76.0	481	78.3
Total	$4{}861$	100.0	614	100.0

Among the 50 directionally consistent migraine-drug pairs, several biologically interpretable clusters emerged. First, aspirin–*GSTP1* (drug rank 26; *GSTP1* higher in cases) was classified as consistent based on a ChEMBL inhibitor annotation; this pair should be interpreted with caution because aspirin’s primary mechanism is COX-1/COX-2 inhibition, and the GSTP1 annotation likely reflects indirect or *in vitro* effects rather than direct pharmacological inhibition. Second, the cardiac glycosides digoxin and digitoxin (drug ranks 126 and 133) were consistent against *ATP1A4* (higher in cases; gene rank 2), which encodes a Na^+^/K^+^-ATPase $\alpha$4 subunit; cardiac glycosides are established Na^+^/K^+^-ATPase inhibitors, making the directionality pharmacologically coherent, though *ATP1A4* itself is supported by only a single-family case report in migraine and should be considered a low-evidence candidate gene rather than an established migraine locus. Third, several GLP-1 receptor agonists (exenatide, liraglutide, semaglutide, lixisenatide; drug ranks 142–146) were mapped against *ALPL* (lower in cases; gene rank 6) via DGIdb annotations; this mapping should be interpreted cautiously because GLP-1 agonists act canonically through GLP1R rather than ALPL directly, and the annotation likely reflects indirect effects on alkaline phosphatase activity rather than direct pharmacological agonism. Fourth, $\alpha _{1}$-adrenergic receptor antagonists including phenoxybenzamine, phentolamine, prazosin, and carvedilol (drug ranks 377–551) were consistent against *ADRA1A* (higher in cases; gene rank 150), where inhibition of elevated adrenergic signaling is mechanistically plausible. Fifth, amitriptyline (drug rank 438; a guideline-supported migraine prophylactic) showed consistent directionality against *AADAC* (higher in cases; gene rank 119) via DGIdb inhibitor annotations.

Directionality filtering materially refined the candidate space by separating broadly recovered compounds from those with stronger mechanistic compatibility. Among migraine-linked or biologically plausible candidates, only a subset remained directionally consistent, while others were directionally inconsistent or unresolved because the mapped action did not align clearly with the inferred disease-associated gene direction. This distinction matters because enrichment alone can recover compounds that are pharmacologically adjacent to migraine biology without necessarily supporting a coherent repurposing hypothesis. Accordingly, we treat directionally consistent pairs as higher-priority computational hypotheses, whereas inconsistent or unresolved pairs should be viewed as context signals rather than actionable leads (Table 7).

**Table 7:** Representative directionally consistent gene–drug pairs from the migraine reference set, defined by mechanistic compatibility between known drug action and the inferred disease-associated direction of the target gene, with drug ranks derived from the integrated gene-to-drug scoring pipeline, gene ranks from the Combined_Score integrated gene-prioritization framework, evidence sources identifying the mechanism-of-action annotation database, and the DGIdb-derived MRPL36 mitochondrial ribosomal inhibitor cluster in rows 3–9 requiring cautious interpretation.

Drug rank	Drug	Gene	Gene rank	Disease direction	Drug action	Evidence source	Approved	Known migraine gene
26	Aspirin	GSTP1	31	Higher in cases	Inhibitor	ChEMBL	Yes	Yes
126	Digoxin	ATP1A4	2	Higher in cases	Inhibitor	DGIdb	Yes	Yes
133	Digitoxin	ATP1A4	2	Higher in cases	Inhibitor	DGIdb	Yes	Yes
142	Exenatide	ALPL	6	Lower in cases	Agonist	DGIdb	Yes	No
144	Liraglutide	ALPL	6	Lower in cases	Agonist	DGIdb	Yes	No
146	Semaglutide	ALPL	6	Lower in cases	Agonist	DGIdb	Yes	No
54	Chloramphenicol	MRPL36	18	Higher in cases	Inhibitor	DGIdb	Yes	No
86	Minocycline	MRPL36	18	Higher in cases	Inhibitor	DGIdb	Yes	No
162	Clarithromycin	MRPL36	18	Higher in cases	Inhibitor	DGIdb	Yes	No
379	Prazosin	ADRA1A	150	Higher in cases	Inhibitor	DGIdb	Yes	Yes
512	Carvedilol	ADRA1A	150	Higher in cases	Inhibitor	DGIdb	Yes	Yes
438	Amitriptyline	AADAC	119	Higher in cases	Inhibitor	DGIdb	Yes	No
257	Empagliflozin	SLC5A2	45	Higher in cases	Inhibitor	DGIdb	Yes	No
222	Glycine	GRIN2B	44	Lower in cases	Agonist	DGIdb	Yes	Yes
497	Loxapine	KCNT1	133	Lower in cases	Activator	DGIdb	Yes	Yes

### Top gene and drug validation panel

To provide a concise summary of the final integrated prioritization, we assembled top-gene and top-drug evidence panels from the *Combined_Score*-ranked gene list and the downstream drug-mapping and directionality outputs. The top-gene panel (Table 8) reports the 10 highest-ranked genes together with their evidence support, inferred disease direction, migraine relevance, and linked druggability information. The top-drug panel (Table 9) reports the 10 highest-ranked drugs with merged target gene information, approval status, and directionality classification.

**Table 8:** Top 10 prioritized genes from the final integrated migraine ranking, ordered by Combined_Score incorporating differential expression, pathway evidence, druggability, and network-hub status, with KnMig indicating a known migraine gene, Tier the confidence tier from the primary composite ranking, NDrugs the number of unique linked drugs, ApprDrug the presence of at least one approved linked drug, and DirBest the strongest directionality support across linked drug pairs.

Rank	Symbol	CombScore	ImpScore	Disease direction	KnMig	Confidence tier	Hits	Tissues	DBs	NDrugs	ApprDrug	DirBest
1	APOBEC3G	0.936	0.435	Higher in cases	No	Tier3_Exploratory	100	29	1	98	Yes	Unclear
2	ATP1A4	0.934	0.531	Higher in cases	Yes	Tier3_Exploratory	18	3	3	24	Yes	Consistent
3	HLA-DQB1	0.933	0.410	Lower in cases	Yes	Tier2_Moderate	32	15	2	55	Yes	Inconsistent
4	PSMB5	0.932	0.413	Lower in cases	No	Tier2_Moderate	40	13	2	33	Yes	Inconsistent
5	SLC1A1	0.932	0.491	Lower in cases	No	Tier2_Moderate	103	15	2	54	Yes	Inconsistent
6	ALPL	0.931	0.412	Lower in cases	No	Tier1_High	35	5	1	96	Yes	Consistent
7	AQP5	0.930	0.419	Lower in cases	No	Tier2_Moderate	42	10	2	29	Yes	Consistent
8	CBL	0.930	0.385	Unclear	No	Tier3_Exploratory	4	2	1	29	Yes	Unclear
9	GRHPR	0.929	0.485	Higher in cases	No	Tier3_Exploratory	206	16	1	2	No	Unclear
10	MRPL21	0.929	0.447	Higher in cases	Yes	Tier2_Moderate	93	31	1	1	No	Unclear

**Table 9:** Top 10 prioritized drug candidates from the final integrated migraine repurposing analysis. Drugs are ranked by *DrugScore*; target genes are merged per drug. KnMig = drug present in curated migraine reference set; NTarg = number of unique target genes among Top 200; Dir = best directionality classification across gene–drug pairs.

Rank	Drug	Phase	KnMig	Approved	NTarg	Target genes	Disease directions	Directionality	Evidence sources
1	Ataluren	APPROVED	No	Yes	4	RPL28, RPS14, RPL12, RPS16	Unclear, lower	Unclear	DGIdb, OpenTargets
2	Metformin	APPROVED	Yes	Yes	2	NDUFV1, NDUFS6	Lower in cases	Inconsistent	OpenTargets, DGIdb
3	Voxelotor	APPROVED	No	Yes	1	HBB	Lower in cases	Unclear	OpenTargets
4	Dibotermin alfa	APPROVED	No	Yes	1	BMPR1B	Higher in cases	Inconsistent	OpenTargets
5	Amantadine	APPROVED	No	Yes	1	GRIN2B	Lower in cases	Inconsistent	OpenTargets
6	Lifitegrast	APPROVED	No	Yes	2	ITGB2, ICAM1	Higher in cases	Consistent	OpenTargets
7	Esketamine	APPROVED	Yes	Yes	1	GRIN2B	Lower in cases	Unclear	OpenTargets, DGIdb
8	Memantine	APPROVED	Yes	Yes	1	GRIN2B	Lower in cases	Unclear	OpenTargets, DGIdb
9	Midostaurin	APPROVED	No	Yes	7	CBL, BMPR1B, CDK3, LATS2, PRKD3, SEPTIN9, PEG3	Mixed	Inconsistent	OpenTargets, DGIdb
10	Apremilast	APPROVED	No	Yes	1	PDE4C	Lower in cases	Inconsistent	OpenTargets

Top gene highlights.

Among the Top-10 integrated-score genes, *ATP1A4* (rank 2; higher in cases; known migraine gene; 24 linked drugs; Consistent directionality) was the most directly disease-relevant, encoding the Na^+^/K^+^-ATPase $\alpha$4 subunit and linking to cardiac glycoside inhibitors including digoxin and digitoxin. *ALPL* (rank 6; lower in cases; Tier 1 confidence; 96 linked drugs; Consistent) was the most druggable top gene, linked to GLP-1 agonists through DGIdb annotations. *HLA-DQB1* (rank 3; lower in cases; known migraine gene; 55 linked drugs) had the broadest drug connectivity but showed inconsistent directionality. *MRPL36* (rank 18; higher in cases; 67 linked drugs; Consistent) was linked to a cluster of antibiotic and antimicrobial drugs through mitochondrial ribosomal inhibition annotations. Among the 49 database-annotated migraine-linked genes present in the Top 200, additional highly ranked examples included *GSTP1* (rank 31; Consistent; aspirin-GSTP1 was the most evidenced approved consistent pair), *GRIN2B* (rank 44; 110 linked drugs; known migraine gene), and *OSGEP* (rank 58; highest composite *Importance:Score* of 0.682; known migraine gene). Several top-ranked genes—including *GRHPR, MRPL21, DHX15*, and *GCDH*—had few or no linked drugs.

Top drug highlights.

Among the Top-10 ranked drugs, lifitegrast (rank 6; approved; targets *ITGB2* and *ICAM1*, both higher in cases; Consistent) was the only approved drug in the Top 10 with directional consistency, acting as an integrin inhibitor compatible with elevated integrin subunit expression. Oleclumab (rank 12; Phase 3; targets *NT5E*; Consistent) and ezatiostat (rank 13; Phase 2; targets *GSTP1*; Consistent) provided the clearest nonapproved consistent candidates. Neramexane (rank 17; Phase 3; targets *GRIN2B* and *CHRNA10*; Consistent) was the highest-ranking investigational drug with a migraine-biology rationale through NMDA receptor modulation. Several top-ranked approved drugs showed directionally inconsistent mappings, including metformin (rank 2; Inconsistent against *NDUFV1* and *NDUFS6*), amantadine (rank 5; Inconsistent against *GRIN2B*), dibotermin alfa (rank 4; Inconsistent against *BMPR1B*), and apremilast (rank 10; Inconsistent against *PDE4C*). Among the Top 20 migraine-reference drugs, aspirin (rank 26; Consistent against *GSTP1*) was the highest-ranking approved drug with directional support, while metformin, esketamine, and memantine appeared at ranks 2, 7, and 8 respectively, consistent with their presence in the migraine drug compendium.

### Biological interpretation of prioritized genes and drug repurposing candidates

The 200 genes prioritized by the integrated G2DR pipeline converged on several biological themes with established or plausible relevance to migraine pathophysiology. Of these, 49 (24.5%) had prior evidence linking them to migraine or closely related headache phenotypes based on database annotation from OMIM, DisGeNET, and HPO, whereas the remainder represent computationally prioritized candidates without established migraine association. These novel signals should be treated as hypothesis-generating outputs of the prioritization pipeline rather than validated migraine targets, and their biological interpretation below is offered to motivate experimental follow-up rather than to make mechanistic claims. Overall, the prioritized genes mapped to pathways related to glutamatergic and ion-channel excitability, mitochondrial and metabolic function, immune and inflammatory signaling, cerebrovascular and extracellular-matrix integrity, and broader neuronal and synaptic maintenance, consistent with the multifactorial biology highlighted by large migraine genetic studies [42, 43].

Among the most interpretable signals, *GRIN2B* and *SLC1A1* supported glutamatergic hyperexcitability and cortical-spreading-depression-related biology [44, 45], while *SCN1B, KCNT1, KCNS2*, and *ATP1A4* pointed to broader disturbance of ion-channel homeostasis and neuronal firing. A second major component comprised mitochondrial and metabolic genes, including *NDUFV1, NDUFS6, MRPL21, MRPL36, TUFM, VARS2, FXN, ETFB, COASY*, and *HAAO*, consistent with longstanding hypotheses that impaired cerebral bioenergetics contributes to migraine susceptibility [46, 47]. Immune and inflammatory signals were also prominent, including multiple HLA-region genes together with *RELA, ICAM1, CLEC7A, LY96, UNC93B1*, and *SERPINE1*, in keeping with evidence that neuroinflammatory and immune-trafficking mechanisms contribute to trigeminovascular sensitization [48–50]. In parallel, neurovascular and extracellular-matrix genes such as *HTRA1, HSPG2, GNAQ, TIE1, EGFL7, LAMB1, F12*, and multiple collagen-related genes supported a vascular-integrity component, consistent with the broader overlap between migraine and cerebral small-vessel or neurovascular disorders [51–53]. Together, these results suggest that the prioritized set reflects convergence across excitability, metabolism, inflammation, and neurovascular maintenance rather than a single dominant molecular pathway.

The downstream drug-mapping analysis likewise recovered a broad migraine-relevant pharmacological landscape rather than only migraine-specific therapies. Using the Top-200 genes, the pipeline identified 3963 unique candidate drugs from 5348 gene–drug evidence rows, and 353 curated migraine-associated drugs were present within the predicted set. Enrichment against the global drug background was strong, indicating that the upstream gene prioritization captures disease-relevant pharmacology even though the output did not capture classical migraine medications. At the top of the ranked list, several recovered migraine-linked drugs were already notable, including metformin (rank 2), esketamine (rank 7), memantine (rank 8), aspirin (rank 26), dexamethasone (rank 48), lidocaine (rank 66), zonisamide (rank 79), indomethacin (rank 85), and acetaminophen (rank 98). Additional recovered migraine-associated or migraine-relevant drugs were present lower in the ranked set, including verapamil (rank 213), valproic acid (rank 253), celecoxib (rank 340), diclofenac (rank 343), amitriptyline (rank 438), topiramate (rank 491), and ergotamine tartrate (rank 521), showing that the pipeline recovered both acute-care and preventive classes as well as broader off-label and mechanism-linked therapies [54–61].

Directionality filtering was particularly useful for separating broadly recovered drugs from those with clearer mechanistic compatibility. Among migraine-linked drugs, aspirin showed consistent directionality at *GSTP1*; amitriptyline was directionally consistent at *AADAC*; and a cluster of antibiotic-derived mappings including chloramphenicol, minocycline, clarithromycin, erythromycin, tigecycline, and tobramycin were directionally consistent through *MRPL36*, although these latter signals should be interpreted cautiously because they arise from mitochondrial ribosomal annotations rather than direct migraine therapeutic use. Several non-reference but biologically interesting candidates were also directionally consistent as computational hypotheses, including lifitegrast at the *ITGB2*–*ICAM1* axis, neramexane at *GRIN2B/CHRNA10*, digoxin and digitoxin at *ATP1A4*, and GLP-1 receptor agonists such as exenatide, liraglutide, and semaglutide, mapped to *ALPL* via an indirect DGIdb annotation (GLP-1 agonists act canonically through GLP1R rather than ALPL directly); notably, GLP-1 agonists have shown promising signals in emerging clinical reports of chronic migraine management [62–65], although all such pairs require direct experimental and clinical validation before any repurposing conclusions can be drawn. By contrast, several high-ranking recovered migraine drugs were directionally inconsistent, including metformin at *NDUFV1/NDUFS6*, dipyridamole at *PDE4C*, carbamazepine, zonisamide, eslicarbazepine, oxcarbazepine, phenytoin, and lidocaine at *SCN5A*, as well as verapamil in its mapped targets. Other prominent recovered drugs, including esketamine, memantine, dexamethasone, indomethacin, acetaminophen, and valproic acid, were retained but remained directionally unresolved. Overall, these results indicate that the G2DR framework recovers a substantial set of migraine-relevant compounds, but that directional filtering is necessary to distinguish broadly associated drugs from those with stronger mechanistic coherence for repurposing. Taken together, the drug-mapping results suggest that G2DR is currently strongest as a framework for broad genetics-anchored translational hypothesis generation. It expands beyond curated disease–target space, recovers migraine-linked pharmacology above background, and benefits from directionality-aware refinement, but it does not yet recover migraine-specific approved therapies with high fidelity. This operating profile is consistent with a framework designed for prioritization and follow-up rather than definitive repurposing recommendation.

## Discussion

We present G2DR as a genotype-first computational prioritization framework for settings in which genotype and phenotype labels are available but matched disease transcriptomics are limited or absent. The principal contribution is not a new TWAS algorithm or a clinically actionable target-identification system, but a modular workflow that converts inherited genetic signal into ranked target and compound hypotheses through multiple complementary evidence layers. In a migraine proof-of-concept, the framework showed internal held-out replication at the gene-prioritization stage, recovered established migraine biology, and produced drug candidate sets enriched for broad migraine-linked pharmacology relative to a global background. However, tiered drug benchmarking showed limited recovery of migraine-specific approved therapies, indicating that the current implementation is better suited to broad pharmacological hypothesis generation than to indication-specific therapy recovery.

Several features of the results are especially informative. First, transcriptome-prediction resources were only moderately concordant, reinforcing that genetically predicted expression is itself model-dependent and that no single reference architecture should be assumed sufficient. Second, component-level benchmarking showed that different evidence layers optimize different objectives: effect-based ranking was strongest for held-out replication, whereas pathway and hub scores more strongly concentrated curated migraine biology. The integrated score should therefore be interpreted as a balanced downstream-selection score rather than as a universally dominant ranking. Third, contextual comparison with Open Targets suggests that G2DR is complementary to established evidence-integration platforms: it broadens the exploratory target space and recovers additional migraine-reference genes outside the Open Targets migraine list, while remaining less concentrated within established disease–target space. The migraine phenotype in this study was based on UK Biobank self-reported migraine status and was not stratified into migraine with aura and migraine without aura. This broad phenotype definition reflects a realistic biobank-style genotype-first setting, but it introduces phenotypic heterogeneity and may dilute subtype-specific genetic signals. Consequently, prioritized genes and compounds should be interpreted as broad migraine-relevant hypotheses rather than subtype-specific mechanisms or subtype-specific therapeutic recommendations. Future applications of G2DR should align phenotype definition with the intended biological or therapeutic question. For subtype-specific prioritization, input cases should ideally be restricted to clinically defined migraine subtypes, including aura-stratified cohorts, electronic health record-confirmed diagnoses, specialist-diagnosed cohorts, medication-supported definitions, or external migraine GWAS/TWAS resources where available. Where stricter phenotype definitions substantially reduce case numbers, this should be reported transparently as a trade-off between phenotype specificity and statistical power. The primary analysis used all available expression-weight tissues because G2DR is intended for genotype-first settings in which the disease-relevant tissue may be uncertain, inaccessible, or unavailable in reference transcriptomic resources. This all-tissue strategy maximized gene coverage and avoided excluding regulatory signals *a priori*, but it was not intended to imply that all tissues are equally relevant to migraine. Trigeminal ganglion is highly relevant to migraine pathophysiology but was not available among the GTEx-derived expression-weight resources used here. To address the possibility that less relevant tissues could introduce noise or dilute tissue-specific signals, we added a tissue-group sensitivity analysis comparing all tissues with brain/CNS, vascular/cardiovascular, and immune/blood tissue subsets. These analyses showed that migraine-relevant tissue groups retained internal held-out replication signal, with curated migraine-gene enrichment strongest in the all-tissue, brain/CNS, and immune/blood groups. These results support the use of all tissues for broad discovery while reinforcing that tissue-level findings should be interpreted as genetically regulated expression-context contributions rather than definitive tissue-specific migraine mechanisms. Future work should incorporate migraine-relevant expression–prediction resources, including trigeminal ganglion, dura, vascular, immune-cell, single-cell, and spatial transcriptomic references when available.

The drug-level results are most informative when interpreted through the tiered benchmark rather than the global-background enrichment alone. While the framework recovered migraine-linked compounds above random expectation, recovery was strongest for broader literature-linked, off-label, and shared-mechanism therapeutic space, whereas migraine-specific approved therapies were under-represented in the top-ranked set. This suggests that the current implementation is better at expanding genetics-anchored translational hypothesis space than at reproducing the most indication-specific modern pharmacology. Directionality filtering further strengthened this interpretation by showing that only a subset of recovered compounds retained clear mechanistic compatibility with the inferred disease-associated gene direction. Together, these findings argue that the strongest present use case for G2DR is structured target and drug-hypothesis prioritization rather than direct inference of therapeutic efficacy.

The causal-support analyses are important precisely because they limit overinterpretation. Single-instrument Mendelian randomization yielded nominal associations only in broader ranked sets, no gene survived multiple-testing correction, and colocalization identified no gene–locus pairs with strong shared-signal support. These findings do not negate the prioritization signal, but they do indicate that the current framework is operating at the level of genetics-anchored candidate ranking rather than causal effector-gene identification. In practical terms, G2DR should therefore be viewed as a hypothesis-prioritization framework whose outputs require orthogonal validation, not as a causal target-discovery engine.

Across cross-validation folds, exact overlap among top-ranked genes was limited, which is expected in a modest, class-imbalanced cohort in which each fold contains only a small number of cases. More importantly, prioritization performance was stable at the signal level, and drug-level rankings were substantially more stable than gene-level identities. This distinction is central to interpreting the framework correctly: for a prioritization method, reproducibility of ranking behavior is more informative than perfect recurrence of specific gene lists. The results therefore support stability of the prioritization signal even when individual top-ranked genes vary across folds.

The study has several important limitations. The migraine cohort is modest and class-imbalanced, which constrains power and likely contributes to both instability in individual gene identities and diffuse significance across highly layered testing structures. Migraine frequently co-occurs with other medical conditions, including psychiatric, cardiometabolic, respiratory/allergic, gastrointestinal, endocrine, inflammatory, and chronic pain-related disorders. We therefore retained a real-world UK Biobank migraine cohort rather than excluding participants with recorded comorbidities. In the matched G2DR cohort, recorded conditions and medication/supplement-related covariates were available for all 733 participants, with no missing values for the summarized variables. Supplementary Table S7 reports the number of cases and controls for the migraine phenotype and for each recorded condition or covariate. Although this design improves the real-world relevance of the framework, it also means that some prioritized genes and drug candidates may reflect shared migraine–comorbidity biology rather than migraine-specific genetic architecture alone. We therefore interpret prioritized genes and compounds as migraine-relevant pharmacological hypotheses for downstream follow-up, not as definitive migraine-specific causal targets or clinically validated migraine therapies.

The framework relies on genetically predicted rather than measured context-specific transcription, and TWAS-style signal remains vulnerable to linkage disequilibrium and co-regulation. Drug mapping is further constrained by database coverage, evidence heterogeneity, and imperfect action-direction annotation. These limitations mean that prioritized genes should be interpreted as genetically supported candidates rather than confirmed causal effectors, and prioritized compounds should be interpreted as computational hypotheses rather than translational recommendations.

Equally informative is what the framework does not yet recover well. The under-representation of serotonergic and CGRP-axis therapies suggests that current public drug–target resources and genetically prioritized gene programs more readily capture shared neurobiological and comorbidity-linked pharmacology than highly indication-specific migraine therapeutics. This operating profile helps explain why broader mechanism-linked candidates are enriched even when migraine-specific approved therapies are sparse in the top-ranked set. Future extensions should therefore focus on stronger causal filters, more explicit perturbational and mechanism-of-action resources, and disease-specific pharmacological annotations that may improve both target specificity and directionality-aware drug ranking.

Overall, G2DR provides a useful foundation for genetics-informed prioritization in genotype-first settings. Its modular design allows independent refinement of transcriptome imputation, gene-level ranking, biological contextualization, and drug mapping, and its present value lies in providing a transparent framework for moving from diffuse inherited signal to a more tractable set of experimentally testable hypotheses. The next critical step is not simply to scale the pipeline, but to strengthen causal attribution, benchmark it more directly against alternative genetics-to-drug workflows, and improve translational specificity through external validation and richer pharmacological annotation. Framed in this way, G2DR is a proof-of-concept prioritization framework for broad genetics-anchored pharmacological hypothesis generation, with clear room for maturation before it can be considered a tool for indication-specific therapy recovery or clinical repurposing recommendation. The held-out test split provides an internal assessment of replication within the same UK Biobank-derived analytical framework, but it does not constitute external validation. The modest number of migraine cases, strong case–control imbalance, and use of a single cohort limit the reliability and external generalizability of the absolute performance estimates. Therefore, the reported ROC–AUC, PR-AUC, and enrichment results should be interpreted as evidence of internal prioritization consistency and biological concordance rather than external clinical generalizability. Future applications of G2DR should validate prioritized targets in independent migraine cohorts, external GWAS or TWAS summary statistics, and disease-relevant cellular, tissue, or pharmacological models. The aggregation strategy also introduces a potential opportunity-burden effect because genes represented across more expression-weight databases, tissues, methods, or folds have more chances to become significant at least once. We addressed this by adding opportunity-corrected and stricter aggregation sensitivity analyses. These analyses confirmed that the headline full-universe ROC–AUC should not be interpreted as strong within-candidate prioritization; rather, it largely reflects separation between genes with discovery evidence and genes without discovery evidence. Within-discovery discrimination was more modest, and the integrated score should therefore be viewed as a pragmatic prioritization heuristic rather than a fully calibrated predictive model. Nevertheless, opportunity-corrected and stricter recurrence analyses retained evidence of internal replication and Top-$K$ enrichment, supporting the framework’s use for hypothesis generation while reinforcing the need for cautious interpretation of individual gene ranks. A further limitation is potential knowledge-source overlap in the drug-level benchmark. Although candidate drugs were generated gene-first from prioritized genes and the migraine drug reference set was assembled phenotype-first from migraine/headache-associated drug resources, both stages can draw on overlapping biomedical knowledge sources. To assess this, we performed a source-overlap sensitivity analysis using the migraine reference list used for the main drug benchmark. The source-overlap analysis identified 4824 unique normalized migraine-reference drugs, of which 307 were supported by at least one prediction-overlap source and 4517 were source-disjoint after excluding reference drugs supported by Open Targets, DGIdb, or ChEMBL. The source-disjoint analysis retained substantial overlap with the ranked predicted drug list, while the prediction-overlap-supported subset captured additional common-source knowledge-base concordance. We therefore interpret the drug-level benchmark as evidence of knowledge-base concordance and broad pharmacological hypothesis generation rather than independent validation of migraine-specific therapy recovery. Full source distributions and source-overlap sensitivity results are provided in Supplementary Tables S9 and S10.

**Key Points**

- G2DR is a genotype-first computational prioritization framework for hypothesis generation in settings where matched disease transcriptomics are unavailable; it is intended to narrow the space for downstream experimental and translational follow-up rather than to provide clinically actionable target recommendations directly.
- The framework integrates genetically predicted gene expression across seven transcriptome-weight resources, multi-method gene-level testing, pathway enrichment, network context, druggability, and multi-source drug–target evidence into a modular and reproducible prioritization pipeline.
- In held-out internal evaluation, discovery-based gene prioritization generalized to test data (ROC–AUC $=0.775$; PR-AUC $=0.475$), while the integrated score provided a balanced operating point for downstream target selection rather than maximizing any single benchmark criterion.
- Drug mapping recovered migraine-linked compounds relative to a global background, but tiered benchmarking showed no recovery of Tier 1 migraine-specific approved therapies within the Top-200 ranked drugs. The strongest signal lay in broader mechanism-linked, off-label, and literature-associated therapeutic space; directionality filtering helped distinguish mechanistically coherent hypotheses from broadly associated candidates.
- All prioritized genes and compounds remain computational hypotheses requiring independent pharmacological, mechanistic, and clinical validation; in its current form, G2DR is best viewed as a proof-of-concept framework for genetics-anchored prioritization with clear directions for improvement in causal attribution and translational specificity.

## Supplementary Material

G2DR_New_Sup_(1)_bbag427

## Data Availability

The genotype and phenotype data used in this study were accessed through the UK Biobank under application ID 50000 (https://www.ukbiobank.ac.uk/) and are subject to UK Biobank access restrictions; researchers may apply for access through the UK Biobank Access Management System. The G2DR framework source code is freely available for non-commercial use at https://github.com/MuhammadMuneeb007/G2DR-A-Genotype-First-Framework-for-Genetics-Informed-Target-Prioritization-and-Drug-Repurposing. Supporting utilities are available at the following repositories: gene phenotype downloader, PhenotypeToGeneDownloaderR (https://github.com/MuhammadMuneeb007/PhenotypeToGeneDownloaderR); gene identifier converter, GeneMapKit (https://github.com/MuhammadMuneeb007/GeneMapKit); drug–disease downloader, DownloadDrugsRelatedToDiseases (https://github.com/MuhammadMuneeb007/DownloadDrugsRelatedToDiseases). All repositories will be maintained for a minimum of two years following publication. Public annotation resources used by the pipeline include STRING (https://string-db.org), Open Targets (https://www.opentargets.org), DGIdb (https://www.dgidb.org), and ChEMBL (https://www.ebi.ac.uk/chembl).
