# Supplementary material for: G2DR: a genotype-first framework for genetics-informed target prioritization and drug repurposing
Source: Brief Bioinform. 2026 Aug 10;27(4):bbag427. doi: 10.1093/bib/bbag427 (PMC13455623; doi:10.1093/bib/bbag427)
Supplement: Supplementary file 1 [file g2dr_new_sup_(1)_bbag427.pdf]

Supplementary Material

G2DR: A Genotype-First Framework for Genetics-Informed Target Prioritization and Drug Repurposing

Muhammad Muneeb[1,2] and David B. Ascher[1,2,*]

[1]School of Chemistry and Molecular Biology, The University of Queensland, Queen Street, 4067, Queensland, Australia

[2]Computational Biology and Clinical Informatics, Baker Heart and Diabetes Institute, Commercial Road, 3004, Victoria, Australia

[*]Corresponding author: David B. Ascher, d.ascher@uq.edu.au

Contents of this Supplementary Material

This document contains seven supplementary methods sections and thirteen supplementary tables. The supplementary methods provide complete mathematical details for the genetically predicted gene expression computation, differential expression analysis, gene prioritization scoring, statistical evaluation framework, directionality annotation criteria, source-overlap sensitivity analysis, opportunity-corrected aggregation analysis, and tissue-group sensitivity analysis that are summarized in the main manuscript.

- **Supplementary Methods S1.** Full mathematical specification of genetically predicted gene expression computation (Equations 1–2) and all eight differential expression and association methods (Equations 3–4). Referenced in the main text Methods section under Genetically Predicted Gene Expression and Differential Expression Analysis.
- **Supplementary Methods S2.** Full scoring formulas for the primary predicted-expression evidence score (DEbase$_g$), including reproducibility (R_g), effect magnitude (E_g), statistical confidence (C_g), PathwayScore propagation, and integrated CoreScore computation. Referenced in the main text Methods section under Gene Prioritization.

- **Supplementary Methods S3.** Full derivation of the hypergeometric enrichment test, permutation-based ranking significance test, and fold-enrichment formula used throughout the Results section. Referenced in the main text Methods section under Gene Prioritization.
- **Supplementary Methods S4.** Full directionality annotation criteria, action vocabulary, and classification rules for gene–drug pair directionality assessment. Referenced in the main text Methods section under Directionality Assessment.
- **Supplementary Methods S5.** Source-overlap sensitivity analysis for the drug-level benchmark. This analysis evaluates whether overlap between candidate drug-generation resources and migraine-reference drug resources contributes to apparent drug-level enrichment.
- **Supplementary Methods S6.** Opportunity-corrected and stricter aggregation sensitivity analysis. This analysis evaluates whether genes with broader database, tissue, method, or fold coverage have more opportunities to become significant and separates full-universe discrimination from within-discovery prioritization.
- **Supplementary Methods S7.** Tissue-group sensitivity analysis. This analysis compares the primary all-tissue discovery strategy with migraine-relevant tissue subsets, including brain/CNS, vascular/cardiovascular, and immune/blood tissue groups.
- **Supplementary Table S1.** Weight stability analysis of the integrated scoring framework across 17 alternative weighting schemes. Referenced in the main text at the Unified Gene Prioritization Framework section.
- **Supplementary Table S2.** Sensitivity analysis of gene prioritization under stricter FDR and effect-size significance thresholds. Referenced in the main text at the Gene Prioritization Robustness section.
- **Supplementary Table S3.** Discovery disease-enrichment for curated migraine genes across all expression-weight databases, tissues, and analytical methods. Referenced in the main text at the Component-Based Recovery section.
- **Supplementary Table S4.** Full comparison of individual evidence components and composite scores across two evaluation universes. Referenced in the main text at the Unified Gene Prioritization Framework section.
- **Supplementary Table S5.** Multi-K overlap evaluation of predicted drugs against the curated migraine reference drug set for top-N input genes ($N = 200$ and $N = 500$) under two evaluation universes. This table provides the primary drug-level benchmark before source-overlap sensitivity analysis.

- **Supplementary Table S6.** External contextual comparison of G2DR with the Open Targets migraine disease-target resource for migraine gene recovery. Referenced in the main text at the Significant Gene Comparison Against Open Targets section.
- **Supplementary Table S7.** Distribution of recorded migraine phenotype, comorbid conditions, and medication/supplement-related covariates in the matched G2DR cohort. Referenced in the main text at the Study Design and Cohort Assembly section.
- **Supplementary Table S8.** Cross-fold stability of fold-specific top-ranked gene lists using pairwise Jaccard similarity, all-fold intersections, fold-wise unions, and random Top-K baselines. Referenced in the main text at the Gene Differential Expression Analysis section.
- **Supplementary Table S9.** Source distribution of the migraine reference drug set and gene-derived predicted drug set. This table reports the number and proportion of unique normalized drugs supported by each biomedical source in the phenotype-derived migraine reference set and the gene-derived predicted drug set.
- **Supplementary Table S10.** Source-overlap sensitivity analysis of the drug-level benchmark. This table reports drug-overlap metrics across the original reference set, prediction-overlap-supported reference drugs, source-disjoint reference drugs, and individual source-ablation scenarios.
- **Supplementary Table S11.** Opportunity-bias and stricter aggregation support summary. This table reports the number of genes retained under opportunity-corrected and stricter evidence rules, including support across folds, databases, tissues, methods, database–tissue–method contexts, and replicated direction.
- **Supplementary Table S12.** Full-universe versus discovery-universe performance under opportunity-corrected and stricter aggregation scores. This table reports ROC-AUC, PR-AUC, and Top-K enrichment metrics separately for the full tested universe and the discovery universe.
- **Supplementary Table S13.** Tissue-group sensitivity analysis of gene-level enrichment and internal held-out replication. This table reports Top-K enrichment for curated migraine genes and held-out test-positive genes across all tissues, brain/CNS tissues, vascular/cardiovascular tissues, and immune/blood tissues.

Supplementary Methods S1. Genetically Predicted Gene Expression and Differential Expression Analysis

Expression prediction. For individual i, gene g, and tissue t, genetically predicted expression was computed as a weighted linear combination of SNP dosages:

$$\widehat{E}_{i,g,t} = \alpha_{g,t} + \sum_{j \in \mathcal{V}_{g,t}} G_{ij}\, w_{j,g,t}, \tag{1}$$

where $G_{ij} \in [0, 2]$ is the allelic dosage of SNP j for individual i, $w_{j,g,t}$ is the pre-trained SNP weight for gene g in tissue t, $\mathcal{V}_{g,t}$ denotes the set of SNPs used by the expression-prediction model for gene g in tissue t, and $\alpha_{g,t}$ is an intercept term. Covariate-adjusted predicted expression was obtained by regressing out sex and the top 10 genetic principal components estimated from the training split:

$$\widehat{E}^{\text{adj}}_{i,g,t} = \widehat{E}_{i,g,t} - \left(\widehat{\gamma}_{g,t} + \widehat{\delta}_{g,t}\,\text{Sex}_i + \sum_{k=1}^{10} \widehat{\beta}_{k,g,t}\,\text{PC}_{ik}\right) + \overline{\widehat{E}}_{g,t}, \tag{2}$$

where $\widehat{\gamma}_{g,t}$, $\widehat{\delta}_{g,t}$, and $\widehat{\beta}_{k,g,t}$ were estimated by ordinary least squares on the training split and applied to validation and test splits without refitting. $\overline{\widehat{E}}_{g,t}$ denotes the training-split mean of $\widehat{E}_{i,g,t}$.

Differential expression methods. For each gene g and tissue t, the case–control mean difference was computed as:

$$\mu^{(1)}_{g,t} = \frac{1}{n_1} \sum_{i:y_i=1} \widehat{E}^{\text{adj}}_{i,g,t}, \qquad \mu^{(0)}_{g,t} = \frac{1}{n_0} \sum_{i:y_i=0} \widehat{E}^{\text{adj}}_{i,g,t}, \qquad \Delta_{g,t} = \mu^{(1)}_{g,t} - \mu^{(0)}_{g,t}. \tag{3}$$

Five differential-expression methods were applied: (i) LIMMA empirical Bayes moderated t-test with effect estimate taken as the fitted phenotype coefficient; (ii) Welch's unequal-variance t-test with effect estimate equal to the mean difference; (iii) OLS regression of expression on phenotype y_i with effect estimate taken as the regression coefficient; (iv) Wilcoxon rank-sum test with effect estimate defined as the median difference; and (v) phenotype-label permutation test using $B = 1{,}000$ random permutations of y_i with an empirical two-sided p-value computed from the permutation distribution of label-shuffled mean differences. Three association-style models treated disease status as the outcome and expression as the predictor. Adjusted expression was standardized to $z_{i,g,t}$ and a logistic model was fitted:

$$\Pr(y_i = 1 \mid z_{i,g,t}) = \frac{1}{1 + \exp[-(\theta_{0,g,t} + \theta_{1,g,t}\, z_{i,g,t})]}, \tag{4}$$

using weighted logistic regression, bias-reduced Firth logistic regression, and a Bayesian logistic approximation. Gene-wise nominal p-values were adjusted using the Benjamini–Hochberg procedure, yielding $\mathrm{FDR}_{g,t}^{(m)}$. Genes were considered significant if $\mathrm{FDR}_{g,t}^{(m)} < 0.1$ and $|\log_2 \mathrm{FC}| \geq 0.5$ for differential-expression methods, or $|\mathrm{Effect}| \geq 0.5$ for association-style methods.

Supplementary Methods S2. Gene Prioritization Scoring

To avoid ambiguity between SNP sets, predicted-expression evidence scores, normalized scoring components, and the final integrated prioritization score, we revised the notation throughout the Supplementary Methods so that $\mathcal{V}_{g,t}$ denotes the SNP set used for expression prediction, DEbase_g denotes the primary predicted-expression evidence score, and $\mathrm{DE}_g^{\mathrm{norm}}$ denotes the normalized form of DEbase_g used in $\mathrm{CoreScore}_g$.

Composite importance score. For each candidate gene $g \in G_{\mathrm{discovery}}$, the primary predicted-expression evidence score is $\mathrm{DEbase}_g = 0.4\, R_g + 0.3\, E_g + 0.3\, C_g$. Reproducibility was quantified as $R_g = 0.6\, \mathrm{norm\_hits}_g + 0.4\, \mathrm{norm\_breadth}_g$, where $\mathrm{norm\_hits}_g$ is the total number of significant occurrences of g across all databases, tissues, methods, and folds normalized to $[0, 1]$ by dividing by the empirical maximum, and $\mathrm{norm\_breadth}_g$ is the number of unique database–tissue–method combinations in which g was significant, similarly normalized. Effect magnitude was computed by standardizing $|\widehat{\psi}_{g,t}^{(m)}|$ within each method m, then aggregating to the gene level as $E_g = 0.7\, f(\bar{e}_g) + 0.3\, f(e_g^{\max})$, where $f(\cdot)$ is a smooth monotonic mapping to $[0, 1]$, $\bar{e}_g$ is the mean standardized absolute effect, and $e_g^{\max}$ is the maximum; if all non-zero effect directions for g were consistent in sign, E_g was multiplied by 1.1 to reward directional stability. Statistical confidence was computed as $C_g = 0.6\,(1 - \mathrm{FDR}_g^{\min}) + 0.4\,(1 - \overline{\mathrm{FDR}}_g)$, where $\mathrm{FDR}_g^{\min}$ is the minimum FDR across all significant results and $\overline{\mathrm{FDR}}_g$ is the mean FDR. Reproducibility was weighted highest (40%) because cross-model replication reduces method-specific artefacts, consistent with TWAS prioritization guidance and the winner's curse literature; effect magnitude and statistical confidence were weighted equally (30% each) to balance biological relevance with statistical support.

PathwayScore propagation. Enrichment was performed using `clusterProfiler::enrichGO`, `clusterProfiler::enrichKEGG`, `ReactomePA::enrichPathway`, and `DOSE::enrichDO` on top-K foreground sets ($K \in \{50, 100, 200, 500, 1000, 2000\}$) with the full tested universe U as background. For each significant enriched term t, a weight was assigned as $\mathrm{weight}(t) = \mathrm{strength}(t) \times \mathrm{robustness}(t)$, where $\mathrm{strength}(t) = -\log_{10}(\mathrm{FDR}_t)$ and $\mathrm{robustness}(t) = n_{\mathrm{hits}}(t)$ counts recurrence of term t as significant across multiple K thresholds and enrichment databases. The PathwayScore for gene g was then $\mathrm{PathwayScore}(g) = \sum_{t:\, g \in \mathrm{Genes}(t)} \mathrm{weight}(t)$, with genes absent from all enriched-term overlap lists assigned $\mathrm{PathwayScore}(g) = 0$.

Integrated CoreScore. The final integrated score combines four evidence layers as a weighted sum: $\mathrm{CoreScore}_g = 0.45 \cdot \mathrm{DE}_g^{\mathrm{norm}} + 0.25 \cdot \mathrm{Path}_g^{\mathrm{norm}} + 0.25 \cdot \mathrm{Drug}_g^{\mathrm{norm}} + 0.05 \cdot \mathrm{Hub}_g^{\mathrm{norm}}$, where $\mathrm{DE}_g^{\mathrm{norm}}$ denotes the normalized form of DEbase_g, the primary predicted-expression evidence score derived from reproducibility, effect magnitude, and statistical confidence.

Differential expression received the highest weight (0.45) because it provides the primary TWAS-derived genetic link between genotype and disease. Pathway and druggability scores received equal moderate weights (0.25 each) to balance biological coherence with therapeutic tractability. Hub score was down-weighted (0.05) because network centrality often reflects pleiotropy, risking prioritization of non-specific, highly connected genes over disease-specific targets. Hub score was derived from STRING-based degree, betweenness, closeness, and eigenvector centrality metrics combined into a single network-priority score. Druggability was assessed by querying DGIdb and ChEMBL for known drug interactions (knowledge-based druggability) and applying `fpocket` to PDB or AlphaFold structures for genes without known drug interactions (structure-based druggability).

Supplementary Methods S3. Statistical Evaluation Framework

ROC-AUC and PR-AUC. Internal held-out replication of the primary predicted-expression evidence score was assessed over the full tested universe $U = 34{,}355$ genes by assigning binary replication labels $y_g = \mathbb{I}(g \in G_{\text{test}})$, where G_{test} comprises genes significant in the held-out test split from the same UK Biobank-derived analytical framework, and using DEbase_g (or CoreScore_g) as the ranking predictor. ROC-AUC and PR-AUC were computed over all genes in U, with genes outside the discovery set assigned a score of zero. The baseline PR-AUC under random ranking equals $T/U = 0.2079$, so the observed PR-AUC of 0.4754 corresponds to a 2.29× lift over random.

Hypergeometric enrichment test. For a top-K list $P_K \subseteq U$ and a positive set of size K^+ (either test-positive genes or curated migraine genes), the hypergeometric p-value is $p_{\text{hyper}} = \Pr(X \geq x)$ for $X \sim \text{Hypergeometric}(N = |U|, K^+, n = |P_K|)$, where $x = |P_K \cap \text{positives}|$ is the observed overlap. Expected overlap under random sampling is $k_{\text{exp}} = |P_K| \times (K^+/|U|)$, and fold-enrichment is $\text{FE} = k_{\text{obs}}/k_{\text{exp}}$.

Permutation test. Statistical significance of the observed ROC-AUC and PR-AUC was evaluated using 1,000 random permutations of gene scores $\{\text{DEbase}_g\}$ while holding labels $\{y_g\}$ fixed, yielding empirical p-values of 9.99×10^{-4} for both metrics (minimum attainable $p = 1/1001$), confirming that the observed ranking lift is not attributable to chance.

Supplementary Methods S4. Directionality Annotation Criteria

Gene direction was assigned from the differential-expression ranking as *higher in cases* (positive mean difference $\Delta_{g,t} > 0$), *lower in cases* (negative mean difference $\Delta_{g,t} < 0$), or *unclear* (conflicting directions across tissues or methods, or no consistent directional signal). Drug action annotations were first extracted from locally aggregated drug–target evidence fields including mechanism-of-action, interaction-type, and directionality fields compiled from Open Targets, DGIdb, and ChEMBL during the drug-mapping step. For pairs lacking explicit local mechanism information, additional annotations were retrieved from ChEMBL by compound identifier and from DGIdb by gene symbol, and were harmonized into a reduced action vocabulary comprising six categories: *inhibitor*, *antagonist*, *agonist*, *activator*, *modulator*, and *unknown*. Each unique gene–drug pair was then classified using the following rules: (i) *directionally consistent* if the drug action was mechanistically compatible with the inferred gene direction — specifically, if the drug is an inhibitor or antagonist against a gene inferred to be higher in cases, or if the drug is an agonist or activator for a gene inferred to be lower in cases; (ii) *directionally inconsistent* if the drug action was opposite to this expectation; and (iii) *unclear* if the gene direction was unresolved, if the drug action was classified as modulator or unknown, or if available annotations were insufficient to support a confident directional interpretation. Directionality was summarized at the level of unique drug–gene pairs to avoid inflation from repeated evidence records for the same pair across multiple databases.

Supplementary Methods S5. Source-overlap sensitivity analysis for the drug-level benchmark

To evaluate whether drug-level enrichment was influenced by overlap between candidate drug-generation resources and migraine-reference drug resources, we performed a source-overlap sensitivity analysis. Candidate drugs were generated gene-first by mapping prioritized genes to drug–target resources, including Open Targets, DGIdb, and ChEMBL. The migraine reference drug set was assembled phenotype-first from migraine/headache-associated drug resources. Although these two steps are conceptually different, both can draw on overlapping biomedical knowledge sources, which may inflate apparent enrichment if the benchmark is interpreted as independent validation.

Using the migraine reference list used for the main drug benchmark, we collapsed the reference set to unique normalized drug names and annotated each reference drug with its supporting source information. This yielded 4,824 unique normalized migraine-reference drugs. We then compared the ranked predicted drug list against multiple reference-set scenarios: the original migraine reference set; reference drugs supported by at least one prediction-overlap source; source-disjoint reference drugs after removing drugs supported by Open Targets, DGIdb, or ChEMBL; and source-specific ablation scenarios in which reference drugs supported by individual overlapping sources were removed. For each scenario and each top-K cutoff, overlap was evaluated using the same hypergeometric framework as the main drug benchmark. This analysis is intended to quantify source dependence and knowledge-base concordance, not to provide independent clinical validation.

Supplementary Methods S6. Opportunity-corrected and stricter aggregation sensitivity analysis

To evaluate whether the discovery aggregation strategy favoured genes with more testing opportunities across expression-weight databases, tissues, methods, or folds, we performed an opportunity-corrected and stricter aggregation sensitivity analysis. All opportunity counts were computed using discovery records only, defined as training and validation records, so that held-out test data were not used during score construction.

For each gene g, the number of valid discovery testing opportunities was defined as:

$$O_g = \sum_{r \in \mathcal{R}_{\text{discovery}}} \mathbb{I}(r \text{ is a valid test for gene } g), \tag{5}$$

where $\mathcal{R}_{\text{discovery}}$ denotes all valid training and validation gene-level tests across folds, expression-weight databases, tissues, and statistical methods. The number of significant discovery hits was defined as:

$$H_g = \sum_{r \in \mathcal{R}_{\text{discovery}}} \mathbb{I}(r \text{ is significant for gene } g), \tag{6}$$

using the same significance criteria as the primary analysis. The opportunity-corrected hit rate was then:

$$\text{HitRate}_g = \frac{H_g}{O_g}. \tag{7}$$

To construct an opportunity-corrected reproducibility score, we percentile-normalized the opportunity-corrected hit rate and the numbers of significant folds, databases, tissues, methods, and database–tissue–method contexts. Let $\widetilde{\text{HitRate}}_g$, $\widetilde{\text{Fold}}_g$, $\widetilde{\text{Database}}_g$, $\widetilde{\text{Tissue}}_g$, $\widetilde{\text{Method}}_g$, and $\widetilde{\text{Direction}}_g$ denote percentile-normalized values for opportunity-corrected hit rate, significant fold support, database support, tissue support, method support, and direction consistency, respectively. The opportunity-corrected reproducibility score was defined as:

$$R_g^{\text{opp}} = 0.50\,\widetilde{\text{HitRate}}_g + 0.15\,\widetilde{\text{Fold}}_g + 0.10\,\widetilde{\text{Database}}_g + 0.10\,\widetilde{\text{Tissue}}_g + 0.10\,\widetilde{\text{Method}}_g + 0.05\,\widetilde{\text{Direction}}_g. \tag{8}$$

We then defined an opportunity-corrected primary predicted-expression evidence score:

$$\text{DEbase}_g^{\text{opp}} = 0.40\,R_g^{\text{opp}} + 0.30\,\widetilde{E}_g + 0.30\,\widetilde{C}_g, \tag{9}$$

where $\widetilde{E}_g$ denotes the percentile-normalized mean absolute effect magnitude across significant discovery records and $\widetilde{C}_g$ denotes percentile-normalized statistical confidence based on the minimum FDR.

In addition to opportunity correction, we evaluated stricter aggregation rules requiring recurrent support across evidence dimensions. These included significance in at least two folds, at least two databases, at least two tissues, at least two methods, at least two database–tissue–method contexts, replicated direction of effect, at least two folds plus at least two contexts, at least two databases plus two tissues plus two methods, and a combined strict rule requiring all recurrence criteria. For each stricter rule, genes failing the criterion were assigned a score of zero, while genes passing the criterion retained the opportunity-corrected score.

Performance was reported separately in three ways. First, full-universe discrimination was evaluated across all tested genes, with genes lacking discovery evidence assigned a score of zero. Second, discovery-universe prioritization was evaluated only among genes in $G_{\text{discovery}}$, providing a stricter test of fine-grained ranking among already-selected candidates. Third, Top-K biological enrichment was evaluated for curated migraine genes and held-out test-significant genes at $K = 50$, 100, and 200.

Supplementary Methods S7. Tissue-group sensitivity analysis

To evaluate whether the all-tissue discovery strategy diluted migraine-relevant biological signal, we performed a tissue-group sensitivity analysis. The primary G2DR analysis used all available expression-weight tissues because the framework is intended for genotype-first settings in which the most relevant disease tissue may be uncertain, inaccessible, or unavailable in reference transcriptomic resources. However, because migraine has neurological, trigeminovascular, vascular, immune/inflammatory, and systemic components, we additionally evaluated tissue-prioritized subsets.

Tissues were grouped into four analysis sets: all available tissues, brain/CNS tissues, vascular/cardiovascular tissues, and immune/blood tissues. The brain/CNS group included brain regions, spinal cord, and tibial nerve. The vascular/cardiovascular group included artery, aorta, and heart tissues. The immune/blood group included whole blood, blood-derived resources, EBV-transformed lymphocytes, and spleen. Trigeminal ganglion, a highly relevant migraine tissue, was not available among the GTEx-derived expression-weight resources used in this study.

For each tissue group, significant genetically predicted expression-associated genes were identified from the discovery splits using the same significance criteria as the primary analysis: FDR < 0.10 and $|\text{effect}| \geq 0.50$, where effect was defined as the available association effect or $\log_2$ fold-change estimate. Within each tissue group, genes were ranked using a transparent group-specific score integrating significant-hit count, mean absolute effect magnitude, and statistical confidence. This tissue-group score was used only for sensitivity analysis and did not replace the primary all-tissue DEbase_g score.

For each tissue group, we evaluated Top-K enrichment for curated migraine genes and held-out test-positive genes at $K = 50$, 100, and 200 using the same hypergeometric framework as the main analysis. We also assessed discovery-versus-test overlap enrichment within each tissue group. This analysis was intended to evaluate whether migraine-relevant tissue groups retained biological and internal replication signal, not to infer definitive tissue-specific causality.

Table S1: Supplementary Table S1. Weight stability analysis of the integrated scoring framework across 17 alternative weighting schemes. Nine reasonable alternative schemes (panel A) and eight extreme single-component stress tests (panel B) were evaluated against the default integrated weights (DE = 0.45, Pathway = 0.25, Drug = 0.25, Hub = 0.05). For each scheme, Spearman ρ against the default ranking, mean Top-100 gene overlap (%), DISC-universe test ROC-AUC (discovery set only, n = 9,305 genes), and FULL-universe test ROC-AUC (all U = 34,355 tested genes) are reported. Across reasonable alternatives, mean ρ = 0.963 (minimum 0.866) and FULL-universe ROC-AUC ranged from 0.775 to 0.776, confirming stability within the biologically justified parameter space. Extreme single-component schemes diverged substantially (mean ρ = 0.682; minimum ρ = 0.292 for hub-only), confirming that the integrated score draws on genuine multi-source signal.

Scheme	DE	Path	Drug	Hub	Spearman ρ	Top-100 overlap (%)	DISC ROC-AUC	FULL ROC-AUC
**(A) Reasonable alternative schemes**								
Default (reference)	0.45	0.25	0.25	0.05	1.000	100.0	0.546	0.776
DE-heavy	0.60	0.15	0.20	0.05	0.978	84.0	0.546	0.776
Pathway-heavy	0.30	0.40	0.25	0.05	0.951	78.0	0.545	0.775
Drug-heavy	0.30	0.25	0.40	0.05	0.962	82.0	0.546	0.775
Equal weights (no hub)	0.33	0.33	0.33	0.00	0.941	77.0	0.545	0.775
Equal weights (with hub)	0.25	0.25	0.25	0.25	0.866	74.0	0.544	0.775
No hub	0.47	0.27	0.27	0.00	0.972	83.0	0.546	0.776
DE + pathway only	0.50	0.50	0.00	0.00	0.948	78.0	0.545	0.775
DE + drug only	0.50	0.00	0.50	0.00	0.955	80.0	0.547	0.776
Pathway + drug balanced	0.40	0.30	0.30	0.00	0.963	82.0	0.546	0.776
**(B) Extreme single-component stress tests**								
DE only	1.00	0.00	0.00	0.00	0.712	58.0	0.675	0.790
Pathway only	0.00	1.00	0.00	0.00	0.651	51.0	0.529	0.549
Drug only	0.00	0.00	1.00	0.00	0.683	55.0	0.511	0.730
Hub only	0.00	0.00	0.00	1.00	0.292	32.0	0.520	0.713
DE + hub only	0.50	0.00	0.00	0.50	0.698	56.0	0.538	0.762
Pathway + hub only	0.00	0.50	0.00	0.50	0.614	48.0	0.524	0.630
Drug + hub only	0.00	0.00	0.50	0.50	0.658	52.0	0.515	0.721
No pathway, no drug	0.95	0.00	0.00	0.05	0.731	60.0	0.658	0.785

Table S2: Supplementary Table S2. Sensitivity analysis of gene prioritization under stricter significance thresholds. For each threshold rule, we report the number of significant genes in the training, validation, and held-out test splits; the size of the discovery set ($G_{\text{discovery}}$); held-out ranking performance (ROC-AUC and PR-AUC evaluated over all $U = 34{,}355$ tested genes); enrichment of discovery genes for held-out test positives (FE test, $T = 7{,}141$); and enrichment for curated migraine genes (FE migraine, $|M \cap U| = 3{,}190$). The primary analysis uses FDR < 0.10 and $|\log_2 \text{FC}| \geq 0.50$ (first row). All stricter settings preserved the main prioritization conclusions: held-out ROC-AUC remained above 0.70, fold-enrichment for test positives ranged from 2.58-fold to 3.20-fold, and fold-enrichment for curated migraine genes ranged from 1.38-fold to 1.41-fold. These results indicate that the main gene-prioritization findings are not driven by permissive significance filtering.

**Threshold rule**	**Train**	**Validation**	**Test**	$G_{\text{discovery}}$	**ROC-AUC**	**PR-AUC**	**FE test**	**FE migraine**
FDR < 0.10, $\lvert\log_2 \text{FC}\rvert \geq 0.50$	1,046	9,107	7,141	9,305	0.7753	0.4754	2.58	1.38
FDR < 0.05, $\lvert\log_2 \text{FC}\rvert \geq 0.50$	958	7,861	5,313	8,135	0.7314	0.3412	2.62	1.39
FDR < 0.10, $\lvert\log_2 \text{FC}\rvert \geq 0.75$	263	6,241	4,599	6,303	0.7339	0.3289	3.20	1.39
FDR < 0.05, $\lvert\log_2 \text{FC}\rvert \geq 0.75$	248	5,709	3,772	5,794	0.7025	0.2497	3.12	1.41

Table S3: Supplementary Table S3. Discovery disease-enrichment for curated migraine genes across expression-weight databases, tissues, and analytical methods. For each component, the discovery set comprises unique genes significant at least once in training and validation within that component. Enrichment is computed against curated migraine genes (M = 3,190) within the analysis universe (U^*). FE denotes fold-enrichment (k_{obs}/k_{exp}). Empirical p-values are from size-matched random gene-set sampling (n_{perm} = 10,000).

**Component**	N_{pred}	k_{obs}	k_{exp}	**FE**	p_{emp}
**(A) Expression-weight databases**					
MASHR	187	40	17.40	2.30	1.00×10^{-4}
JTI	104	18	9.68	1.86	6.799×10^{-3}
FUSION	9,146	1,156	851.16	1.36	1.00×10^{-4}
EpiXcan	34	5	3.16	1.58	2.06×10^{-1}
CTIMP	16	2	1.49	1.34	4.51×10^{-1}
TIGAR	16	2	1.49	1.34	4.48×10^{-1}
UTMOST	3	0	0.28	0.00	1.00
**(B) Tissues (top 10 by discovery enrichment)**					
Brain Amygdala	312	57	29.04	1.96	1.00×10^{-4}
Minor Salivary Gland	315	57	29.31	1.94	1.00×10^{-4}
Whole Blood	381	65	35.46	1.83	1.00×10^{-4}
Adrenal Gland	399	67	37.13	1.80	1.00×10^{-4}
Esophagus Gastroesophageal Junction	419	70	38.99	1.80	1.00×10^{-4}
Cells EBV-transformed Lymphocytes	366	61	34.06	1.79	1.00×10^{-4}
Brain Anterior Cingulate Cortex BA24	309	51	28.76	1.77	2.00×10^{-4}
Brain Spinal Cord Cervical C-1	312	51	29.04	1.76	1.00×10^{-4}
Lung	502	80	46.72	1.71	1.00×10^{-4}
Artery Coronary	380	60	35.36	1.70	1.00×10^{-4}
**(C) Analytical methods**					
Weighted Logistic	9,299	1,189	865.39	1.37	1.00×10^{-4}
Bayesian Logistic	9,264	1,184	862.13	1.37	1.00×10^{-4}
Welch t-test	917	115	85.34	1.35	6.00×10^{-4}
Linear Regression	19	2	1.77	1.13	5.36×10^{-1}
LIMMA	19	1	1.77	0.57	8.53×10^{-1}
Firth Logistic	3	0	0.28	0.00	1.00

Table S4: Supplementary Table S4. Comparison of individual evidence components and composite scores across two evaluation universes. Full universe (U = 34,355 genes): non-discovery genes receive score = 0; measures pipeline-level separation of discovery-relevant genes from the entire tested space. **Discovery universe** (n = 9,305 genes): discovery set only (train ∪ val); measures within-set ranking quality. T ROC = test-replication ROC-AUC; T PR = test-replication PR-AUC; K ROC = known-migraine ROC-AUC; K PR = known-migraine PR-AUC. Top-200 FE_{test} and FE_{mig} denote fold-enrichment relative to random expectation. Expected Top-200 overlap: full-universe test = 41.57, full-universe migraine = 18.57; disc-universe test = 107.36, disc-universe migraine = 25.56. All raw components were percentile-normalized before ranking.

	AUC metrics				Top-200 test		Top-200 migraine	
**Ranking**	**T ROC**	**T PR**	**K ROC**	**K PR**	**Obs**	**FE**	**Obs**	**FE**
**(A) Full universe (U = 34,355 genes; non-discovery genes scored as 0)**								
Significance only	0.776	0.477	0.557	0.107	146	3.51	25	1.35
Effect only	0.790	0.526	0.558	0.111	145	3.49	37	1.99
Primary composite ($DEbase_g$)	0.775	0.475	0.557	0.109	156	3.75	36	1.94
Pathway only	0.549	0.265	0.520	0.108	150	3.61	66	3.55
Hub only	0.713	0.396	0.572	0.135	114	2.74	64	3.45
Druggability only	0.730	0.408	0.566	0.112	116	2.79	25	1.35
Direct target evidence only	0.629	0.320	0.543	0.109	110	2.65	57	3.07
Drug-link count only	0.771	0.440	0.561	0.111	105	2.53	26	1.40
Integrated	0.776	0.472	0.562	0.118	140	3.37	49	2.64
**(B) Discovery universe (n = 9,305 genes; train ∪ val only)**								
Significance only	0.548	0.593	0.505	0.131	146	1.36	25	0.98
Effect only	0.675	0.663	0.519	0.140	145	1.35	37	1.45
Primary composite ($DEbase_g$)	0.543	0.590	0.510	0.135	156	1.45	36	1.41
Pathway only	0.529	0.564	0.536	0.162	150	1.40	66	2.58
Hub only	0.520	0.551	0.629	0.210	114	1.06	64	2.50
Druggability only	0.511	0.544	0.569	0.148	121	1.13	29	1.14
Direct target evidence only	0.511	0.543	0.542	0.152	110	1.02	58	2.27
Drug-link count only	0.505	0.539	0.553	0.141	109	1.02	30	1.17
Integrated	0.546	0.585	0.562	0.161	140	1.30	49	1.92

Table S5: Supplementary Table S5. Multi-K overlap evaluation of predicted drugs against the curated migraine reference drug set ($|\mathcal{R}| = 4{,}824$ normalized drugs). Results are shown for top-N input genes ($N = 200$ and $N = 500$) under two evaluation universes. **ALL**: global background of 139,597 drugs; hypergeometric enrichment test is valid. **PREDICTED**: returned candidate pool only; FE values below 1.0 reflect the fact that the reference drug set exceeds the returned candidate pool in size, making standard hypergeometric enrichment interpretation inapplicable — AUROC and AUPRC are the appropriate metrics in this frame. Within-set performance: for $N = 200$, AUROC = 0.8004 and AUPRC = 0.3528 (353 curated migraine drugs among 3,963 predicted drugs); for $N = 500$, AUROC = 0.8152 and AUPRC = 0.3311 (527 curated migraine drugs among 7,981 predicted drugs).

N	Universe	K	$\lvert\mathcal{U}\rvert$	$\lvert\mathcal{R}\rvert$	Overlap	Prec@K	Rec@K	F1@K	Expected	FE
**Top-$N = 200$ input genes**										
200	ALL_DRUGS	20	139,597	4,824	5	0.250	0.001036	0.002064	0.691	7.235
200	ALL_DRUGS	50	139,597	4,824	20	0.400	0.004146	0.008207	1.728	11.575
200	ALL_DRUGS	100	139,597	4,824	51	0.510	0.010572	0.020715	3.456	14.758
200	ALL_DRUGS	200	139,597	4,824	89	0.445	0.018449	0.035430	6.911	12.877
200	ALL_DRUGS	500	139,597	4,824	205	0.410	0.042496	0.077010	17.278	11.865
200	PREDICTED	20	3,963	4,824	5	0.250	0.001036	0.002064	24.345	0.205
200	PREDICTED	50	3,963	4,824	20	0.400	0.004146	0.008207	60.863	0.329
200	PREDICTED	100	3,963	4,824	51	0.510	0.010572	0.020715	121.726	0.419
200	PREDICTED	200	3,963	4,824	89	0.445	0.018449	0.035430	243.452	0.366
200	PREDICTED	500	3,963	4,824	205	0.410	0.042496	0.077010	608.630	0.337
**Top-$N = 500$ input genes**										
500	ALL_DRUGS	20	139,597	4,824	8	0.400	0.001658	0.003303	0.691	11.575
500	ALL_DRUGS	50	139,597	4,824	20	0.400	0.004146	0.008207	1.728	11.575
500	ALL_DRUGS	100	139,597	4,824	53	0.530	0.010987	0.021527	3.456	15.337
500	ALL_DRUGS	200	139,597	4,824	93	0.465	0.019279	0.037022	6.911	13.456
500	ALL_DRUGS	500	139,597	4,824	213	0.426	0.044154	0.080015	17.278	12.328
500	PREDICTED	20	7,981	4,824	8	0.400	0.001658	0.003303	12.089	0.662
500	PREDICTED	50	7,981	4,824	20	0.400	0.004146	0.008207	30.222	0.662
500	PREDICTED	100	7,981	4,824	53	0.530	0.010987	0.021527	60.444	0.877
500	PREDICTED	200	7,981	4,824	93	0.465	0.019279	0.037022	120.887	0.769
500	PREDICTED	500	7,981	4,824	213	0.426	0.044154	0.080015	302.218	0.705

Table S6: Supplementary Table S6. External contextual comparison of G2DR with the Open Targets migraine disease-target resource for migraine gene recovery. The full G2DR analysis was evaluated across the complete ranked gene universe ($n = 9{,}305$ genes). The target-filtered G2DR analysis was restricted to genes represented in the Open Targets migraine target space ($n = 823$ genes). Overall recovery is shown against the curated migraine reference set ($M = 3{,}190$ genes), together with Top-K precision before and after filtering. The substantially higher precision of standalone Open Targets (Top-50: 92.00%) reflects its disease-curated, pre-filtered design rather than a direct performance comparison with G2DR. G2DR operates across a wider gene universe and recovered 725 curated migraine reference genes absent from Open Targets entirely, indicating that the two resources are complementary rather than directly competitive.

**Metric**	**G2DR (all genes)**	**G2DR (target-filtered)**	**Open Targets**
Ranked / returned genes	9,305	823	2,376
Reference genes recovered	1,189	464	1,228
Reference recovery (%)	37.27	14.55	38.50
Shared with Open Targets	823	823	–
Shared with Open Targets and reference	464	464	464
Reference genes recovered but absent from Open Targets	725	0	–
Top-50 precision (%)	22.00	46.00	92.00
Top-100 precision (%)	22.00	50.00	95.00
Top-200 precision (%)	23.00	55.50	92.50
Top-500 precision (%)	27.60	56.60	92.00

Table S7: Supplementary Table S7. Distribution of recorded migraine phenotype, comorbid conditions, and medication/supplement-related covariates in the matched G2DR cohort. All listed variables were assessed in the full matched cohort of 733 participants, with no missing values. For non-migraine rows, "cases" denotes participants positive for the listed condition or covariate and "controls" denotes participants negative for that condition or covariate; these are not migraine case–control labels.

Condition / covariate	Cases, n	Controls, n	Total samples	Missing, n
Migraine phenotype	53	680	733	0
Asthma	115	618	733	0
Osteoarthritis	94	639	733	0
High cholesterol	81	652	733	0
Irritable bowel syndrome	78	655	733	0
Hypothyroidism / myxoedema	71	662	733	0
Hay fever / allergic rhinitis	64	669	733	0
Gastro-oesophageal reflux / gastric reflux	46	687	733	0
Cholesterol-lowering medication	117	616	733	0

Table S8: Supplementary Table S8. Cross-fold stability of fold-specific top-ranked gene lists. For each cross-validation fold, training and validation records were combined and genes were ranked using fold-local predicted-expression evidence. Pairwise Jaccard similarity was computed across all fold pairs for Top-50, Top-100, Top-200, Top-500, and Top-1000 ranked gene lists. Random baselines were estimated from 1,000 random Top-K gene sets sampled from the train-validation gene universe.

Top-K	Mean Jaccard	SD Jaccard	Mean overlap	Mean overlap fraction	All-fold intersection	Union across folds	Random mean Jaccard
50	0.0663	0.0168	6.2	0.1240	2	206	0.0007
100	0.0618	0.0163	11.6	0.1160	3	418	0.0015
200	0.0675	0.0164	25.2	0.1260	6	822	0.0029
500	0.0771	0.0118	71.5	0.1430	11	1976	0.0073
1000	0.0929	0.0081	169.9	0.1699	22	3751	0.0148

Table S9: Supplementary Table S9. Source distribution of migraine reference drugs and gene-derived predicted drugs. The table reports the number and percentage of unique normalized drugs supported by each biomedical source in the phenotype-derived migraine reference set and the gene-derived predicted drug set. The expanded migraine reference file contained 4,824 unique normalized migraine-reference drugs, while the gene-derived predicted drug list contained 3,963 unique normalized drugs. Sources overlapping with candidate drug generation, including Open Targets, DGIdb, and ChEMBL, were used to define prediction-overlap-supported and source-disjoint reference subsets for the source-overlap sensitivity analysis.

Set	Source	Drugs supported	Percentage of set
Reference migraine drugs	Open Targets	162	3.36%
Reference migraine drugs	DGIdb	0	0.00%
Reference migraine drugs	ChEMBL	145	3.01%
Reference migraine drugs	CTD	3,996	82.84%
Reference migraine drugs	ClinicalTrials.gov	710	14.72%
Reference migraine drugs	SIDER	49	1.02%
Reference migraine drugs	DrugCentral	86	1.78%
Reference migraine drugs	Any prediction-overlap source	307	6.36%
Predicted gene-derived drugs	Open Targets	74	1.87%
Predicted gene-derived drugs	DGIdb	1,405	35.45%
Predicted gene-derived drugs	ChEMBL	2,663	67.20%
Predicted gene-derived drugs	Any prediction-overlap source	3,963	100.00%

Table S10: Supplementary Table S10. Source-overlap sensitivity analysis of the drug-level benchmark. The table evaluates whether drug-level enrichment is influenced by overlap between drug-mapping resources and migraine-reference drug resources. The original reference set was compared with prediction-overlap-supported reference drugs, source-disjoint reference drugs, and source-specific ablation scenarios. Common-source scenarios evaluate knowledge-base concordance, whereas source-disjoint scenarios provide a conservative anti-circularity sensitivity analysis. For each scenario, overlap between the ranked predicted drug list and the corresponding reference set was evaluated at Top-100 and Top-200 using the global drug universe. These results should not be interpreted as independent clinical validation.

Analysis	K	Reference size	Overlap	Expected	Fold enrichment	Precision	Hypergeom. p
Original reference	100	4,824	51	3.39	15.04	0.510	1.64×10^{-47}
Original reference	200	4,824	89	6.78	13.13	0.445	4.65×10^{-75}
Prediction-overlap-supported reference	100	307	8	0.22	37.08	0.080	6.72×10^{-11}
Prediction-overlap-supported reference	200	307	11	0.43	25.49	0.055	1.06×10^{-12}
Source-disjoint reference	100	4,517	43	3.17	13.54	0.430	1.98×10^{-37}
Source-disjoint reference	200	4,517	78	6.35	12.28	0.390	1.10×10^{-62}
Exclude Open Targets-supported reference drugs	100	4,662	51	3.28	15.57	0.510	3.01×10^{-48}
Exclude Open Targets-supported reference drugs	200	4,662	89	6.55	13.58	0.445	2.46×10^{-76}
Exclude ChEMBL-supported reference drugs	100	4,679	43	3.29	13.08	0.430	8.50×10^{-37}
Exclude ChEMBL-supported reference drugs	200	4,679	78	6.58	11.86	0.390	1.52×10^{-61}
Only ChEMBL-supported reference drugs	100	145	8	0.10	78.50	0.080	1.64×10^{-13}
Only ChEMBL-supported reference drugs	200	145	11	0.20	53.97	0.055	2.75×10^{-16}

Table S11: Supplementary Table S11. Opportunity-bias and stricter aggregation support summary. Opportunity counts were computed from discovery records only, defined as training and validation records. The table reports how many genes remained under increasingly strict evidence rules designed to reduce potential inflation from genes with broader model coverage or more testing opportunities.

**Evidence rule**	**Number of genes**
Genes with valid discovery opportunities	34,278
Genes with at least one significant discovery hit	9,305
Genes significant in at least two folds	4,203
Genes significant in at least two databases	157
Genes significant in at least two tissues	3,415
Genes significant in at least two methods	9,264
Genes significant in at least two database–tissue–method contexts	9,270
Genes with replicated direction of effect	6,893
Genes significant in at least two folds and at least two contexts	4,203
Genes significant in at least two databases, two tissues, and two methods	152
Genes passing all strict criteria	60

Table S12: Supplementary Table S12. Full-universe versus discovery-universe performance under opportunity-corrected and stricter aggregation scores. Full-universe metrics evaluate separation between discovery-supported genes and the broader tested background, whereas discovery-universe metrics evaluate fine-grained ranking among already-selected candidate genes. This distinction is important because full-universe ROC-AUC can be inflated by assigning zero scores to non-discovery genes.

Score / rule	Nonzero genes	Full test ROC-AUC	Full test PR-AUC	Discovery test ROC-AUC	Discovery test PR-AUC	Top-100 migraine FE	Top-100 test FE
Original DEbase$_g$	9,305	0.7753	0.4754	0.5431	0.5904	2.26	3.80
Opportunity-corrected hit rate only	9,305	0.7719	0.4358	0.5117	0.5338	0.22	2.12
Opportunity-corrected reproducibility	9,305	0.7752	0.4688	0.5417	0.5809	0.97	3.32
Opportunity-corrected DEbase$_g^{opp}$	9,305	0.7779	0.4767	0.5660	0.5923	0.97	3.27
Strict: at least two folds	4,203	0.6546	0.3672	0.5838	0.5924	1.08	3.13
Strict: at least two databases	157	0.5056	0.2145	0.5026	0.5400	1.72	3.37
Strict: at least two tissues	3,415	0.6322	0.3524	0.5854	0.5987	1.40	3.37
Strict: at least two contexts	9,270	0.7770	0.4760	0.5659	0.5922	0.97	3.27
Strict: replicated direction	6,893	0.6860	0.3854	0.4881	0.5603	0.86	3.51
Strict: all criteria	60	0.5023	0.2108	0.5015	0.5385	1.19	2.26

Table S13: Supplementary Table S13. Tissue-group sensitivity analysis of gene-level enrichment and internal held-out replication. The table compares the primary all-tissue strategy with migraine-relevant tissue subsets, including brain/CNS, vascular/cardiovascular, and immune/blood tissue groups. For each tissue group, genes were ranked using discovery-split significant genetically predicted expression evidence, and Top-100 enrichment was evaluated for curated migraine genes and held-out test-positive genes. The analysis assesses whether tissue-prioritized subsets retain biological enrichment and internal replication signal, but it should not be interpreted as evidence of definitive tissue-specific causality.

Tissue group	No. tissues	Discovery genes	Test-positive genes	Top-100 migraine observed	Top-100 migraine FE	Migraine p	Top-100 test FE	Test p
All tissues	64	9,305	7,141	19	2.04	2.03×10^{-3}	2.97	4.90×10^{-19}
Brain/CNS	14	3,398	2,594	20	1.76	8.32×10^{-3}	6.69	3.82×10^{-42}
Vascular/cardiovascular	7	1,683	1,140	17	1.37	1.09×10^{-1}	11.37	9.86×10^{-49}
Immune/blood	5	1,062	770	22	1.77	5.01×10^{-3}	14.19	5.00×10^{-50}

End of Supplementary Material
